# Liver‐originated small extracellular vesicles with TM4SF5 target brown adipose tissue for homeostatic glucose clearance

**DOI:** 10.1002/jev2.12262

**Published:** 2022-09-05

**Authors:** Jae Woo Jung, Ji Eon Kim, Eunmi Kim, Hyejin Lee, Haesong Lee, Eun‐Ae Shin, Jung Weon Lee

**Affiliations:** ^1^ Department of Pharmacy, College of Pharmacy Seoul National University Seoul Republic of Korea; ^2^ Research Institute of Pharmaceutical Sciences, College of Pharmacy Seoul National University Seoul Republic of Korea; ^3^ Interdisciplinary Program in Genetic Engineering Seoul National University Seoul Republic of Korea

**Keywords:** brown adipose tissue, glucose transporter, hepatic sEVs, protein–protein interaction, tetraspanin

## Abstract

Transmembrane 4 L six family member 5 (TM4SF5) is involved in chronic liver disease, although its role in glucose homeostasis remains unknown. TM4SF5 deficiency caused age‐dependent glucose (in)tolerance with no link to insulin sensitivity. Further, hepatic TM4SF5 binding to GLUT1 promoted glucose uptake and glycolysis. Excessive glucose repletion caused hepatocytes to secrete small extracellular vesicles (sEVs) loaded with TM4SF5 (hep‐sEV^Tm4sf5^), suggesting a role for sEV^Tm4sf5^ in glucose metabolism and homeostasis. Hep‐sEV^Tm4sf5^ were smaller than sEV^Control^ and recruit proteins for efficient organ tropism. Liver‐derived sEVs, via a liver‐closed vein circuit (LCVC) using hepatic TM4SF5‐overexpressing (*Alb‐*Tm4sf5 TG) mice (liv‐sEV^Tm4sf5^), improved glucose tolerance in *Tm4sf5* ^−/−^ KO mice and targeted brown adipose tissues (BATs), possibly allowing the clearance of blood glucose as heat independent of UCP1. Taken together, hep‐sEV^Tm4sf5^ might clear high extracellular glucose levels more efficiently by targeting BAT compared with hep‐sEV^Control^, suggesting an insulin‐like role for sEV™^4SF5^ in affecting age‐related metabolic status and thus body weight (BW).

## 1 INTRODUCTION

We have an interest in understanding how aging is associated with pathological conditions such as cancer and metabolic diseases (Niccoli & Partridge, 2012). Nine different hallmarks of aging have been proposed, such as deregulated nutrient sensing and mitochondrial dysfunction, and the sensing of blood glucose levels is the most conserved aging‐controlling pathway (Lopez‐Otin et al., 2013). Elevated blood glucose levels may threaten our lives, by promoting excess body weight (BW) and obesity with type 2 diabetes (Apovian et al., 2019), followed by progression from diabetes mellitus to cancer progression (Supabphol et al., 2021). Thus, understanding or identifying the molecules and pathways involved in blood glucose regulation would clinically benefit patients.

Glucose is the most important cellular energy source that sustains life; its availability should be constantly monitored, and its maintenance is mandatory for survival (Pozo & Claret, 2018). The liver protects the body from hypoglycaemia via numerous biochemical pathways, including gluconeogenesis and glycogenolysis, to provide glucose during periods of starvation, which are mediated by glucagon that is expressed and secreted by the α cells of the pancreas. Meanwhile, hyperglycaemia is regulated by the pancreatic β‐cell‐mediated secretion of insulin, which activates glucose uptake in muscle and adipose tissue by translocating glucose transporter 4 (GLUT4) to the plasma membrane and initiating the energy storage cascade of glycolysis and glycogenesis in the liver (Anyamaneeratch et al., 2015; Haythorne & Ashcroft, 2021). The hypothalamus controls systemic glucose homeostasis and peripheral metabolic processes in the liver, muscle, fat and pancreas (Lin & Accili, 2011; Pozo & Claret, 2018). Given the importance of controlling blood glucose levels for energy utilization and metabolic activity, understanding or identifying the molecules and pathways involved in blood glucose clearance would clinically benefit patients with diabetes, obesity and cancer and even impact longevity. Because pathological phenotypes related to abnormal blood glucose levels are observed more in older adults, the relationship between chronic regulation of blood glucose and aging may be critical for managing quality of life (Brewer et al., 2016). The cross‐talks between liver and brown adipose tissue (BAT) or muscle may provide insights into how homeostatic regulation of blood glucose levels might be achieved by insulin and other related hormones. Exosomes or small extracellular vesicles (sEVs) may also be functional for the aspects.

Exosomes or sEVs are nanovesicles secreted from all type of cells, ranging from 10 to 250 nm in diameter, which transfer proteins, lipids, bioactive molecules, DNA and RNA for cell–cell communication (Gurung et al., 2021). Exosomes were early considered components of cellular waste but have been recently considered active in cell–cell communication following specific mechanisms of exosome genesis and function (Jadli et al., 2020). They originate from intraluminal vesicles and are formed by the inward budding of the membrane of multivesicular bodies (MVBs) that either lead to proteolytic degradation by fusion with lysosomes or are released as exosomes/sEVs into the extracellular environment following fusion with the plasma membrane (Akers et al., 2013). Therefore, for hormones like insulin, sEVs may play roles in the modulation of blood glucose levels after being packaged with certain glucose‐modulating components.

Transmembrane 4 L six family member 5 (TM4SF5) is a tetraspanin containing four transmembrane domains, two extracellular loops and cytosolic N‐ and C‐terminal tails that are expressed in the liver (Lee, 2015). TM4SF5 is a highly *N*‐glycosylated and palmitoylated protein localized to the plasma and lysosomal membranes (Jung et al., 2019; Kim et al., 2017). The lysosomal localization of TM4SF5, together with mTOR, leads to the activation of mTORC1 via lysosomal arginine sensing and transport into the cytosol (Jung et al., 2019). In addition, TM4SF5 has been shown to be involved in non‐alcoholic fatty liver disease (Ryu et al., 2021). The metabolism of amino acids and other nutrients (e.g. fatty acids and carbohydrates) is regulated by TM4SF5 expression in hepatocytes (Kim et al., 2021; Lee et al., 2022). Because TM4SF5 in hepatocytes appears to regulate metabolic pathways via intracellular traffic to different membranous compartments (Jung et al., 2019; Kang et al., 2014), TM4SF5 is likely loaded on sEVs and plays roles in the metabolic cross‐talks between the liver and BAT.

Here we investigated the mechanism by which hepatic TM4SF5 regulates blood glucose levels, as it relates to insulin sensitivity and BW. We rationalized that TM4SF5 may play roles in hepatocyte‐derived sEVs to modulate blood glucose levels. We found that TM4SF5 deficiency led to glucose intolerance without any influence by insulin, presumably leading to the healthy regulation of BW, which was achieved by sEV^Tm4sf5^ derived from hepatocytes targeting BAT for efficient energy usage and blood glucose clearance.

## 2 MATERIALS AND METHODS

### 2.1 Experimental design

TM4SF5 in hepatocytes is involved in the development of non‐alcoholic liver disease via roles in metabolic pathways. In this study, the roles of TM4SF5 in the clearance of blood glucose were explored in homeostatic status and organ‐crosstalk from liver to BAT axis manner.

### 2.2 Cell culture

SNU‐761, NCI‐H727, HEK293A, Huh7 and SNU449 cell lines were purchased from the Korean Cell Line Bank (Seoul National University, Seoul, Korea), HEK293FT cells were purchased from Thermo Fisher Scientific (R7007, Waltham, MA) and AML12 cells were obtained from the American Type Culture Collection (Manassas, VA). Huh7, HEK293A and HEK239FT cells were maintained in DMEM (SH30243.01, Hyclone, Logan, UT), and Hep3B and SNU449 cell lines were grown in RPMI1640 (SH30027.01, Hyclone) supplemented with 10% FBS (F0600, GenDEPOT, Barker, TX) and penicillin/streptomycin (P/S, CA005, GenDEPOT). AML12 cells were maintained in DMEM/F12 (SH30023.01, Hyclone) supplemented with 10% FBS, 1× insulin‐transferrin‐selenium (ITS, 41400045, Gibco, Waltham, MA), and 40 ng/ml dexamethasone (265005, Sigma‐Aldrich, St. Louis MO). All cells were grown at 37°C in 5% CO_2_ and passaged every 3–4 days, according to the manufacturer's instructions, but for no more than 20 passages. In case, cells were transfected with siRNA or shRNA against a control sequence or human TM4SF5 or mouse GLUTs for 48 h (Table 1).

**TABLE 1:** siRNA or shRNA targeting sequences against TM4SF5 or GLUTs

Number of siRNA targeting sequence against human *TM4SF5*		Sequence (5′→ 3′)
siRNA‐NS		GenePharma Negative control #A06001
siTM4SF5 #4	Sense	CCA UCU CAG CUU GCA AGU C
	Anti‐sense	GAC UUG CAA GCU GAG AUG G
siTM4SF5 #7	Sense	CCUCCU GCU GGU ACC UAA U
	Anti‐sense	AUU AGG UAC CAG CAG GAG G
siTM4SF5 #8	Sense	GCU UGC AAG UCU GGC UCA U
	Anti‐sense	AUG AGC CAG ACU UGC AAG C

Number of shRNA targeting sequence against human *TM4SF5*	Sequence (5′→ 3′)
shRNA‐NS	CCTAAGGTTAAGTCGCCCTCGCTCGAGCGAGGGCGACTTAACCTTAGG
shTM4SF5 #4	CCGGCCATCTCAGCTTGCAAGTCCTCGAGGACTTGCAAGCTGAGATGGTTTTTG
shTM4SF5 #5	CCGGTGGACCCAGATGCTTAATGCTCGAGCATTAAGCATCTGGGTCCATTTTTG
shTM4SF5 #12	CCGGTGGACCCAGATGCTTAATGAACTCGAGTTCATTAAGCATCTGGGTCCATTTTTG

Number of siRNA targeting sequence against mouse *GLUTs*		Sequence (5′→ 3′)
siRNA‐NS		GenePharma Negative control #A06001
siGLUT1	Sense	GCC UCU GCUGCU CAG UGU CAU CUU CAU
	Anti‐sense	AUG AAG AUG ACA CUG AGC AGC AGA GGC
siGLUT2	Sense	GGA CAA ACG GAA GGA CAA
	Anti‐sense	UUG UCC UUC CGU UUG UCC
siGLUT4	Sense	GAC UGG AAC ACU GGU CCU AGC UGU AU
	Anti‐sense	AUA CAG CUA GGA CCA GUG UUC CAG UC

### 2.3 Small extracellular vesicle (sEV) purification

sEVs were purified from cultured media, sera and liver‐circulated biofluids. For cultured media and liver‐circulated fluids, RPMI 1640 supplemented with 10% ultrafiltered (UF) FBS was used to eliminate bovine‐derived sEVs. UF‐FBS was achieved by filtering FBS through a 100‐kD MW cut‐off centrifugal concentrator (Sartorius, Göttingen, Germany) followed by 0.2‐μm sterile filtration. For glucose starvation, RPMI 1640 without glucose (US Biological, Salem, MA) supplemented with 10% dialysed (3‐kD MW cut‐off, VWR, Radnor, PA) UF‐FBS was used (i.e. d/UF‐FBS). Cells were grown in a growth medium for 2 days, washed twice with PBS and incubated in an appropriate volume of 10% UF media for 2 days to collect sEVs. The hepatocytes were cultured in glucose‐null (or ‐depleted) culture media for 50 min or the indicated time. They were then supplied with glucose at 11 mM (in RPMI‐1640 culture medium for SNU449 cells) or 25 mM (in DMEM for Huh7 cells) for 10 min (in the case of no‐indication) or the indicated periods before sEV purification. Crude cultured media were centrifuged at 2000 *× g* and 4°C for 10 min and passed through a 0.2‐μm sterile filter to remove cells and debris. Cleared samples were centrifuged again at 10,000 × *g* and 4°C for 45 min to remove large vesicles and concentrated to 500 μl for culture media and liver‐circulated biofluids, or 100 μl for sera, using a 50‐kD MW cut‐off centrifugal concentrator (Sartorius). Concentrated samples were loaded onto a size‐exclusion chromatography column (qEV original 35 nm or qEV single 35 nm, iZON, Christchurch, New Zealand) that was pre‐equilibrated with PBS at room temperature. A void volume of 3 ml (qEV original) or 1 ml (qEV single) was discarded, and 1.5 ml (qEV original) or 0.6 ml (qEV single) of the sEV‐containing fractions were pooled for further analysis.

### 2.4 Cell proliferation rate analysis

Huh7 or AML12 cells were seeded in 6‐well plates at 5 × 10^4^ cells/well in either normal 10% FBS‐, UF‐FBS or d/UF‐FBS for 24 ∼ 96 h and counted again after the indicated times. UF‐FBS was prepared, as explained above, to deplete sEVs in the serum, and d/UF‐FBS was prepared to remove the glucose in the UF‐FBS during the analysis of extracellular glucose‐dependent sEV release from the hepatocytes or livers (Eitan et al., 2015; Witwer et al., 2013). The 24‐h proliferation rate was calculated and the rates of UF‐ or d/UF‐FBS‐treated cells were normalized to the control (i.e. cells cultured in normal exosome‐containing FBS). Statistical comparisons were performed using by two‐way analysis of variance.

### 2.5 DNA plasmids

Plasmid constructs used included TM4SF5‐STrEP^®^, mouse Tm4sf5‐STrEP^®^ in pEXPR‐IBA‐103 (IBA Lifesciences, Goettingen, Germany), HA‐TM4SF5, HA‐mouse Tm4sf5, HA‐GLUT1, HA‐GLUT2, HA‐GLUT3, HA‐GLUT4 and HA‐GLUT9, which were cloned into the pCMV‐HA‐N vector (Clontech). GLUT1 was cloned from a mixed cDNA library of different cell lines into the pCMV‐HA‐N vector using the *Sal*I/*Not*I enzyme site; the following primers were used: forward 5′‐AAA GTC GAC CAT GGA GCC CAG CAA GAA G‐3′ and reverse 5′‐TTT GCG GCC GCT CAC ACT TGG GAA TCA GC‐3′. GLUT2, 3, 4 and 9 were sub‐cloned from cDNAs purchased from Addgene; GLUT2, 4 and 9 were gifts from Wolf Frommer (Addgene plasmid 18086, 18087 and 18730) and GLUT3 was a gift from David Sabatini (Addgene plasmid 72877).

### 2.6 Immunoprecipitation (IP)

One million HEK293FT cells were plated onto 100‐mm culture dishes. After 16 h, cells were transfected with the indicated cDNA constructs using polyethyleneimine (with linear MW of ∼25,000, sc‐360988, Santa Cruz Biotechnology). After 48 h, cells were washed twice with ice‐cold PBS and lysed with IP lysis buffer (40‐mM HEPES, pH 7.4, 150‐mM NaCl, 1‐mM EDTA and 0.5% Triton X‐100) containing protease inhibitors (P3100, GenDEPOT). Lysates were centrifuged at 12,000 × *g* for 15 min at 4°C. The supernatant was saved, and 30 μl of a 50% Strep‐conjugated agarose bead slurry (20353, Thermo Fisher Scientific) was added. The mixture was incubated at 4°C with rocking (50 rpm) for 4 h. Beads were washed three times with IP wash buffer (40‐mM HEPES, pH 7.4, 500‐mM NaCl, 1‐mM EDTA and 0.5% Triton X‐100) and once with ice‐cold PBS. Proteins were eluted with 2× Laemmli sample buffer (50 μl) by boiling at 95°C for 5 min.

### 2.7 Western blots

Size‐exclusion‐purified sEVs were concentrated to 50 μl using a 50‐kD MW cut‐off centrifugal concentrator (Sartorius). Concentrated sEVs were lysed in 1× RIPA buffer and normalized using a BCA assay kit (23225, Thermo Fisher Scientific). Cell lysates were also prepared in the same way. Western blots were performed following a standard protocol. Primary antibodies used (at 1:1000 dilution) included ALIX (#2171), CD54 (#4915), FLAG (#2368), UCP1 (#14670), COX IV (#4850), TOM20 (#42406), mTor (#2983) and pS^2448^mTor (#2976) from Cell Signaling Technology; CD63 (sc‐5275) and β‐actin (sc‐47778) from Santa Cruz Biotechnology; HA from BioLegend (901515) and Strep‐HRP (2‐1509‐001) from IBA Lifesciences. The polyclonal anti‐TM4SF5 antibody was generated by immunizing rabbits with the sequence peptide for the human TM4SF5 C‐terminal (CGG‐^190^RKKQDTPH^197^) or the long extracellular loop (aa 118‐130) region (Pro‐Sci, Poway, CA).

### 2.8 Immunofluorescence

Cover glasses were coated with 10‐μg/ml fibronectin (35600, BD Biosciences, San Jose, CA) in PBS at 4°C for 16 h and briefly rinsed three times with PBS. Twenty thousand cells were plated on precoated cover glasses in 12‐well plates for 16 h. Cells were then transfected with the indicated cDNA constructs using Lipofectamine 3000 (Invitrogen, Grand Island, NY). After 48 h of transfection, the cover glasses were fixed with ice‐cold 99% methanol for 10 min and blocked with 1% BSA in PBS for 1 h at room temperature (RT). Primary antibodies [anti‐FLAG (NB600‐344, Novus Biologicals, Littleton, CO), anti‐HA (901515, Bio Legend), anti‐GLUT1 and anti‐GLUT4 (12939, 2213, Cell Signaling Technology)] were diluted in 1% BSA in PBS and incubated with the cover glasses for 16 h at 4°C. The cover glasses were then washed three times with PBS and incubated with fluorescence‐conjugated secondary antibodies [Alexa Fluor 488 (goat: A11055, mouse: A21202 and rabbit: A21206) and Alexa Fluor 555 (goat: A21432, mouse: A31570 and rabbit: A31572) from Invitrogen] in PBS for 1 h at RT. The cover glasses were then washed three times with PBS for 5 min each and mounted on glass slides using ProLong™ Gold Antifade (P36930, Invitrogen) with or without a 1‐min incubation with DAPI. Cells were randomly visualized using a Nikon Eclipse Ti microscope and a C2 confocal system; representative images were analysed using NIS‐Elements software (Nikon, Melville, NY).

### 2.9 Transmission electron microscopy

For negative staining, sEVs were purified from media (five 150‐mm dishes) conditioned for 48 h with Huh7 (RPMI‐1640 containing 10% UF‐FBS) or AML12 (DMEM/F12 containing 10% UF‐FBS, 1× ITS and 40‐ng/ml dexamethasone) cells transfected with pCDNA3‐v5‐TM4SF5‐APEX2 plasmid. Purified sEVs were concentrated to 50 μl using a 50‐kD MW cut‐off centrifugal concentrator (Sartorius). Five microliters of concentrated sEVs were spotted on the glow‐discharged carbon grid (CF‐200CU, electron microscopy sciences, Hatfield, PA) for 1 min and rinsed with distilled water. sEVs on the grid were stained with 5% uranyl acetate for 20 s and dried for imaging. Images were obtained using a 120‐kV transmission electron microscope (TEM, Talos L120C, FEI, Hillsboro, OR). For cryogenic electron microscopy (cryo‐EM), sEVs were purified in the same way as for negative staining but using twenty 150‐mm plates. Three microliters of the samples were prepared for cryo‐EM using the Vitrobot system (FEI). APEX2 staining was adopted and modified from Martell et al. (2017). Briefly, 0.5 mg/mL 3,3′‐diaminobenzidine (DAB, Sigma‐Aldrich), and 10 mM H_2_O_2_ (Sigma‐Aldrich) were added to 1× PBS at kept at 4°C. Ten microliters of the DAB solution were added to 50 μl of concentrated exosome solution and incubated for 1 min. Five hundred microliters PBS were added to the solution, and excess DAB was removed by concentrating the solution to 50 μl using a 50‐kD MW cut‐off centrifugal concentrator. Five microliters of 2% (w/v) OsO_4_ in cold PBS were added to the sEV samples and incubated for 5 min prior to negative staining or cryo‐EM.

### 2.10 Mice

WT, *Tm4sf5* ^−/−^ and *Alb*‐Tm4sf5 transgenic (TG) C7BL/6N mice were housed in a specific pathogen‐free room with controlled temperature and humidity. All animal procedures were performed in accordance with the procedures of the Seoul National University Laboratory Animal Maintenance Manual and with IRB approvals from the Institute of Laboratory Animal Resources, Seoul National University (SNU‐191214‐1, SNU160527‐10, SNU‐161108‐10‐1, SNU‐220509‐1 and KIRAMS 2022‐0081). *Tm4sf5* knockout C57BL/6N mice (*Tm4sf5^−/−^*) were generated as described previously (Ryu et al., 2021). The exon 1 region (CCACCTGGACGGACGGCAACCTCAGC) of *Tm4sf5* was excised using CRISPR RGEN technology. *Tm4sf5^−/+^* heterozygotes from the F1 litter mates were bred to generate *Tm4sf5^−/−^* homozygotes. Genotyping for KO mice was performed using forward (5′‐CCA AGC CTC CCA CCT GTT‐3′) and reverse (5′‐GCT CCA GCA TTC TCA CCA‐3′) primers to observe a PCR product of 241 bps. Albumin promoter‐conjugated mouse Tm4sf5‐(FLAG)_3_ (*Alb*‐Tm4sf5) TG C57BL/6N mice were generated at Macrogen (Seoul, Korea) by germline transmission. Genotyping of *Alb*‐Tm4sf5 TG mice was performed using forward (5′‐CAG CTT GGC TTG AAC TCG TTC‐3′) and reverse (5′‐CAA TTC CTG GAC ACA GCA CCA‐3′) primers to observe a PCR product of 689 bps. The transgenic mice were backcrossed with stock C57BL/6N mice every five generations. Next, the experimental mice were maintained under temperature controlled (25°C), free‐moving conditions on a 14‐h dark, 10‐h light cycle. They were group‐housed throughout the study with 2∼5 mice per cage. Age‐matched C57BL/6N male mice had access to tap water and a normal chow diet (NCD) ad libitum. The littermates of WT, KO or *Alb*‐Tm4sf5 TG mice (isolated from different litters) were used in experiments after randomized assignment following genotyping. Adeno‐associated virus type 8 (AAV8), used to induce mouse Tm4sf5 or the control vector (Cont), was manufactured by Virovek (Hayward, CA) and intravenously injected at a dose of 2 × 10^10^ vg/mouse into 8‐week‐old mice (*n* = 10), and experiments were performed 2 weeks after injection. The AAV8‐injected liver tissues were further processed for immunohistochemistry or immunoblots. WT or KO mice (*n* = 25) have been followed longer than 27 months under normal chow ad libitum, with the determination of BWs every month and survival rates. In parallel, another set of the littermates (*n* = 10) was analysed for *Tm4sf5* mRNA levels using collected liver tissue, 1 week after IPGTT and IPITT at 3, 6, 12 and 18 months. Additionally, the *Tm4sf5* mRNA levels were determined in primary hepatocytes or BAT from WT (*n* = 5∼6) or KO mice (*n* = 7) at 6 months of age. The primers for qRT‐PCR were 5′CAC CTG GAC GGA CGG CAA CC‐3′ for the forward and 5′‐CAG CAA CCT GCA CCG CAG CAG‐3′ for the reverse reaction.

### 2.11 High‐carbohydrate diet experiment

Eight‐week‐old WT or *Tm4sf5^−/−^* C57BL/6N mice (*n* = 10) were fed a high‐carbohydrate diet (HCD) (70% kcal carbohydrate diet, HCD, TD.98090, Teklad/Harlan, Madison, WI) ad libitum for 10 weeks. BW was measured every week, and glucose tolerance and insulin tolerance tests were performed on the 8th week.

### 2.12 Glucose/insulin tolerance test

Mice were fasted for either 16 h (before the glucose tolerance test, GTT) or 6 h (before the insulin tolerance test, ITT) with water supplied in a new cage. After fasting, BWs and initial glucose levels were recorded using a scale and glucometer (One touch ultra, Johnson and Johnson, New Brunswick, NJ), respectively. After these initial measurements, 2‐g/kg D‐glucose (G8270, Sigma‐Aldrich) in PBS (for GTT) or 0.5‐U/kg recombinant insulin (91077C, Sigma‐Aldrich) in PBS (for ITT) were injected intraperitoneally (*n* = 10). Blood glucose levels were measured from the tail vein at time 0 and then every 30, 60 and 120 min after the injection.

### 2.13 Liver‐closed vein circuit (LCVC)

Mice were anaesthetized using a cotton swab soaked in 2 ml of 30% isoflurane (Terrell™, Piramal, PA) in polyethylene glycol 200 (8.07483, Sigma‐Aldrich) and maintained under anaesthesia during the entire procedure. Mice were immobilized on a surgical board with a heating pad (42°C), and the lower ventral abdomen was opened using surgical scissors. Gastric organs were moved to the medial side to visualize the hepatic portal vein, which was isolated from the surrounding fat using forceps, and a loose knot was placed proximally to the branched vein. An auto‐retracting 22‐G catheter (381812, BD Biosciences) was inserted into the portal vein right below the entry to the liver. The ligature was tightened, and the catheter was connected to a peristaltic pump via silicon tubing and attached to PBS containing 0.5‐mM EGTA at 42.0°C under oxygen. The pump was set at a flow rate of 0.8–1.2 ml/min, depending on the condition of the liver. To visualize the hepatic inferior vena cava, the sternum was cut open. The surrounding fat was removed from the inferior vena cava and an auto‐retracting 22‐G catheter was inserted from the proximal side. A ligature was secured to the catheter and connected to a waste receptacle. The opened abdomen was covered with cotton gauze soaked in PBS, and infrared lighting was used to maintain body temperature. After a 30‐min flush with a PBS/EGTA solution, the peristaltic pump was connected to a solution of RPMI‐1640 containing 10% UF‐FBS under oxygen. The catheter in the inferior vena cava was connected to the tubing, which returned the biofluids back to the original pool. The circulated fluids were kept at RT to prevent clogs in the tubing. Media/biofluids were circulated for 1–2 h. The liv‐sEV^Tm4sf5‐FLAG^ prepared via LCVC from the *Alb*‐Tm4sf5 TG mice were intravenously injected into the KO mice (10‐week‐old male mice; *n* = 10), whereas PBS was parallelly injected into age‐matched WT mice. One day later, IPGTTs were performed for the animals without (for basal IPGTT) or with the PBS or liv‐sEV^Tm4sf5^ injection. PBS was parallelly injected into WT mice to distinguish the effects mediated by exogenous liv‐sEV^Tm4sf5‐FLAG^ from those by endogenous sEV^Tm4sf5^ possibly available in the WT mice. The parallel injection of PBS (instead of no‐injection) was also performed to null‐out the injection‐mediated stress for blood glucose levels during the effect comparison.

### 2.14 ^14^C‐labelled glucose clearance examination

Liv‐sEV^Tm4sf5‐FLAG^ prepared via LCVC from *Alb*‐Tm4sf5‐FLAG TG mice (*n* = 10) were injected at 1 × 10^9^ sEVs/mouse in 200 μl of PBS into the tail veins of KO (*n* = 6) male mice at 16 weeks of age. Two hours later, WT, KO mice (*n* = 5) and the sEV^Tm4sf5‐FLAG^‐injected KO mice were intraperitoneally injected with ^14^C‐glucose [^14^C(U)‐glucose, 40 μCi/kg, Lot# 842‐162‐270‐A‐20180731‐SBA; Moravek Inc., Brea, CA]. ^14^C‐glucose [40 μCi/kg, 10 ml/kg in ethanol/H_2_O (9:1)] was pre‐mixed with cold glucose (1 g/kg, 10 ml/kg in normal saline). The retro‐orbital blood samples (50 μl each) from the mice were collected at 5, 10, 30, 60, 90 and 120 min following the ^14^C‐glucose injection, centrifuged at 14,000 × *g* for 10 min, and transferred to a glass vial. The samples were then added with 15 ml of ultima gold solution (Perkin Elmer), vortexed and stabilized in a dark area before determining β‐counts for radiolabelled‐glucose levels in the samples using a liquid scintillation counter (LSC; Perkin Elmer, Hopkinton, MA). The experiments and manipulation of the radiolabelled wastes were performed at the Korea Radioisotope Center for Pharmaceuticals (KRICP, Seoul, Korea) in the Korea Institute of Radiological & Medical Sciences (KIRAMC) according to their regulations (SNU‐220509‐1 and KIRAMS 2022‐0081).

### 2.15 BAT thermogenesis analysis

The rectal and BAT temperatures were measured for each animal (16‐week‐old WT or KO mice; *n* = 4∼6) using a traceable double thermometer (T22‐147‐943; LKlab Korea, Namyangju, Korea) with selectable resolutions of 0.1°C/1°C for a 0.8‐ to 1.0‐s sampling time. The rectal and interscapular BAT temperatures were measured simultaneously for each mouse, every minute (0∼5 min) after 20% isoflurane anesthesia following cold exposure for 3 h. The temperature sensor for BAT was positioned to the interscapular BAT immediately after a skin incision. The difference [ΔT(°C)] between the rectal (as a control temperature for the body) and BAT read‐outs in each animal was considered the BAT‐based thermogenic effects depending on TM4SF5 expression. The ΔT(°C) values of each animal group at each time point were statistically compared using the Mann–Whitney *U* test.

### 2.16 Seahorse analysis

A Seahorse XFe24 analyzer (Agilent, Santa Clara, CA) was used to measure the extracellular acidification rate (ECAR) and oxygen consumption rate of the cells. Cells were seeded on an XF24 culture plate at a density of 2 × 10^4^ cells/well, and a glycolytic stress test and real‐time ATP rate assay were performed, as described by the manufacturer. For the glucose sensitivity assay, cells were starved for glucose for 1 h and then treated with the indicated concentrations of glucose and 1‐mM oligomycin (75351, Sigma‐Aldrich). For primary brown adipose cells, mouse pre‐brown adipose cells were plated on an XF24 culture plate and differentiated as described above. On differentiation day 7, cells were washed twice with Seahorse basal medium and incubated with 4 × 10^8^ sEVs/well in Seahorse basal medium. Brown adipose cells and sEVs were incubated at 37°C for 1 h prior to the experiment.

### 2.17 Glucose uptake assay

Glucose uptake assays were performed using the Glucose Uptake‐Glo™ Assay (J1343, Promega, WI), according to the provided protocol. Briefly, for liver cell lines, 1 × 10^4^ cells were plated in a 96‐well plate and transfected with the indicated plasmid DNAs or siRNAs (Table 1) using Lipofectamine 3000 or RNAiMAX (Invitrogen), respectively. After a 48‐h transfection, cells were washed twice with PBS and incubated in 50‐μl glucose‐free RPMI‐1640 containing 1‐mM 2‐DG. Cells were incubated for 20 min at RT before adding 25 μl of the stop solution. Luciferase buffer was prepared 1 h before the experiment, according to the manufacturer's instructions. One hundred microliters of luciferase buffer were added to the cells prior to incubation for 1 h. Samples were transferred to an opaque 96‐well plate, and luminescence was measured. For primary brown adipose cells, mouse pre‐brown adipose cells were plated on a 96‐well plate and differentiated as described above. On differentiation day 7, 4 × 10^8^ AML12‐sEVs were added to the cells and incubated at 37°C under 5% CO_2_ for 90 min as described above.

### 2.18 Nanoparticle tracking analysis

After size‐exclusion chromatography, purified sEVs were analysed using NTA (Nanosight LM10, Malvern Panalytical, Malvern, UK). Samples were diluted with PBS at appropriate concentrations for analysis (serum ∼1:100, cultured media ∼1:10). For each sample, representative images were acquired for 60 s. Random triplicate images were obtained. Vesicle size and concentration were calculated based on their average values using Nanosight NTA software.

### 2.19 Tunable resistive pulse sensing

Purified sEVs were analysed by tunable resistive pulse sensing (qNano Gold, iZON). A nanopore size of 40–320 nm (NP100, iZON) was used. PBS, the coating solution and calibration beads were from iZON. Nanopores were coated and equilibrated with the supplied buffers. Nanopores were manually stretched to approximately 45 mm, and a pressure of 1–10 mbar was applied to achieve 500–1000 particles/min. A baseline current was set to 140 nA to apply 0.2–1.0 V.

### 2.20 Primary brown adipose cell culture

BAT was obtained from 8‐week‐old male mice and kept in ice‐cold PBS until needed. Once needed, the PBS was removed, and the tissue was finely minced using a sterile razor blade. Minced tissue was transferred to a 15‐ml conical tube containing 1 ml of sterile‐filtered 123.0‐mM NaCl, 5.0‐mM KCl, 1.3‐mM CaCl_2_, 5.0‐mM glucose, 100.0‐mM 2‐hydroxyethylpiperizine‐N′‐2‐ethanesulfonic acid (HEPES), 4% BSA, 1× penicillin/streptomycin (P/S), 1‐mg/ml collagenase II and 1‐ml PBS. The tissue was digested for 30 min at 37°C with gentle agitation, and 0.4‐ml FBS was added to stop the digestion before passing through a 100‐μm cell strainer. The digested tissue was centrifuged at 600 × *g* for 5 min at RT. The supernatant was removed, and the pellet was resuspended in DMEM/F12 supplemented with 15% FBS and 1× P/S. The cells were incubated in media on a 100‐mm dish at 37°C under 5% CO_2_ for 24 h. Cells were then washed twice with PBS, and new media were added. Media were changed every day until the cells reached approximately 80% confluence. Cells were detached using Accutase (A6964, Sigma‐Aldrich) and replated (96‐well plate, Seahorse XP24 plate, etc.). Cells were cultured until confluence prior to differentiation in differentiation media [0.5‐mM 3‐isobutyl‐l‐methylxanthine (I5879, Sigma‐Aldrich), 1‐μM dexamethasone (D4902, Sigma‐Aldrich), 1‐μg/ml insulin (I0516, Sigma‐Aldrich), 1‐μM rosiglitazone (557966, Sigma‐Aldrich), 1‐nM 3,3′,5‐triiodo‐L‐thyronine (T3, T2877, Sigma‐Aldrich), 15% FBS and 1× P/S in DMEM/F12] for 48 h. After incubation, cells were further differentiated in DMEM/F12 supplemented with 1‐μg/ml insulin, 1‐nM T3, 15% FBS and 1× P/S for 6 days with fresh media changes every other day.

### 2.21 Intravital imaging of organ incorporation of near‐infrared‐tracer‐labelled sEVs

Purified sEVs were labelled with near‐infrared dye using the ExoGlow™‐Vivo EV labelling kit (EXOGV900A‐1, System Biosciences, Palo Alto, CA), following the manufacturer's instructions. Unbound dye was removed using 20% sucrose‐cushioned ultracentrifugation at 100,000 × *g* for 70 min. Labelled sEVs were resuspended in 100‐μl PBS and intravenously injected into the tail veins of mice at approximately 4 × 10^8^ sEVs/mouse. Three days prior to the injection, mice were switched to a defined diet (AIN‐93G, Teklad) to eliminate any autofluorescence from the chow diet. Incorporation of the near‐infrared‐dye‐labelled sEVs to the organs were visualized using the IVIS spectrum in vivo imaging system with emission 800 nm/excitation 745‐nm filters. Identical organ images were normalized to the same degree of radiant efficiency for comparison.

### 2.22 Proteomics

Purified sEVs from hepatocytes or the LCVC were concentrated to 50 μl using a 50‐kD MW cut‐off concentrator. Fifteen microliters of sample were reduced in 5‐mM TCEP (75259, Sigma‐Aldrich) and 10‐mM ammonium bicarbonate (AMBIC, A6141, Sigma‐Aldrich) for 40 min at 56°C. Seven microliters of 55‐mM iodoacetamide (I1149, Sigma‐Aldrich) in 50‐mM AMBIC were then added and incubated for 30 min in a dark chamber at RT for alkylation. The sample was reduced again in 3‐μl of 42.5‐mM TCEP in 50‐mM AMBIC for 10 min in the dark at RT. Six volumes of ice‐cold acetone were added, vortexed and incubated overnight at −20°C for precipitation. The sample was centrifuged at 15,000 *× g* for 10 min at 4°C, and the supernatant was removed. The pellet was resolubilized in 27‐μl 50‐mM AMBIC. For trypsin digestion, 500‐ng trypsin (UI288664, Thermo) in 50‐mM AMBIC was added; for chymotrypsin digestion, 500‐ng chymotrypsin (90056, Thermo) in 50‐mM AMBIC and 10‐mM CaCl_2_ were added and incubated for 16 h at 37°C. The second digestion was performed using 100‐ng enzyme in 80% acetonitrile (900667, Sigma‐Aldrich) for 3 h at 37°C. To stop the digestion, formic acid (UH287092, Thermo) was added to a final concentration of 5%. The sample was then dried under vacuum (CVE‐2200, Eyela, Singapore) and resolubilized in 10‐μl 0.1% trifluoroacetic acid (TFA, T6508, Sigma‐Aldrich). A peptide clean‐up was performed using a C‐18 ZipTip (ZTC18S, Millipore), following the manufacturer's protocol. Peptides were eluted with 10‐μl 80% acetonitrile, 20% MS‐grade water (115333, Sigma‐Aldrich) and 0.1% TFA. The eluted sample was mixed with α‐cyano‐4‐hydroxycinnamic acid (C8982, Sigma‐Aldrich) and spotted onto a MALDI plate for MALDI‐TOF analysis (rapifleX, Bruker, MA). All reagents were reconstituted and diluted with MS‐grade water or its equivalent.

### 2.23 Proteomics analysis

The MALDI‐TOF spectrum was smoothed, baseline corrected, and peaks were identified using mMass software. A contaminant spectrum was prepared using a parallel sample without sEVs. The contaminant spectrum was removed by mass filtering with a tolerance of 20 ppm. Peptide sequence matching was performed using variable modifications of all cysteines, N‐terminus carbamidomethylation and oxidation of methionines and tryptophans with a tolerance of 20 ppm. For the peak analysis of common peptides, peaks were selected from identified peaks with a 0.05‐Da allowance. The intensities of the common peaks were compared using principal component analysis in the Clustvis software with vector scaling and SVD with imputation for principal component calculations. DAVID (v6.8, http://david.ncifcrf.gov/) was used to perform the functional annotation of the identified proteins based on the Kyoto Encyclopedia of Genes and Genomes pathway database (http://www.genome.jp/kegg/pathway.html). STRING (version 11.0, http://string‐db.org/) and Cytoscape software (v3.8.2) were used to analyse the protein–protein interaction network.

### 2.24 Immunohistochemistry

BAT was analysed using hematoxylin and eosin staining and/or immunohistochemistry (Grosset et al., 2019) with anti‐HA tag (#3724), anti‐mTor (#2983) or anti‐pS^2448^mTor (#2976, Cell Signaling Tech.) antibodies. Tissue sections were incubated with the antibodies overnight at 4°C, and binding was detected using the VECTASTAIN^®^ ABC‐HRP kit (Vector Laboratories, Burlingame, CA). Mayer's hematoxylin (Sigma‐Aldrich) was used for counterstaining nuclei. Ten random images per slide were obtained using a digital slide scanner (MoticEasyScan, Motic, British Columbia, Canada).

### 2.25 Statistical analysis

For statistical analyses, all data points were tested for normality using either the D'Agostino‐Pearson or Shapiro–Wilk normality test. The Student's *t* test or one‐way ANOVA was performed for those passing a normality test using the Dunnett's multiple comparison post‐test. The Mann–Whitney or Kruskal–Wallis test was performed for those not passing a normality test. For samples involving the analysis of two factors for significance, a two‐way ANOVA was performed, and *P* values of single sources of variation are indicated on the graph. All statistical calculations were performed using Prism software (GraphPad, San Diego, CA), and *P* values less than 0.05 were considered statistically significant.

## 3 RESULTS

### 3.1 *Tm4sf5* deficiency modulates glucose tolerance without a significant impact by insulin resistance

**FIGURE 1:**
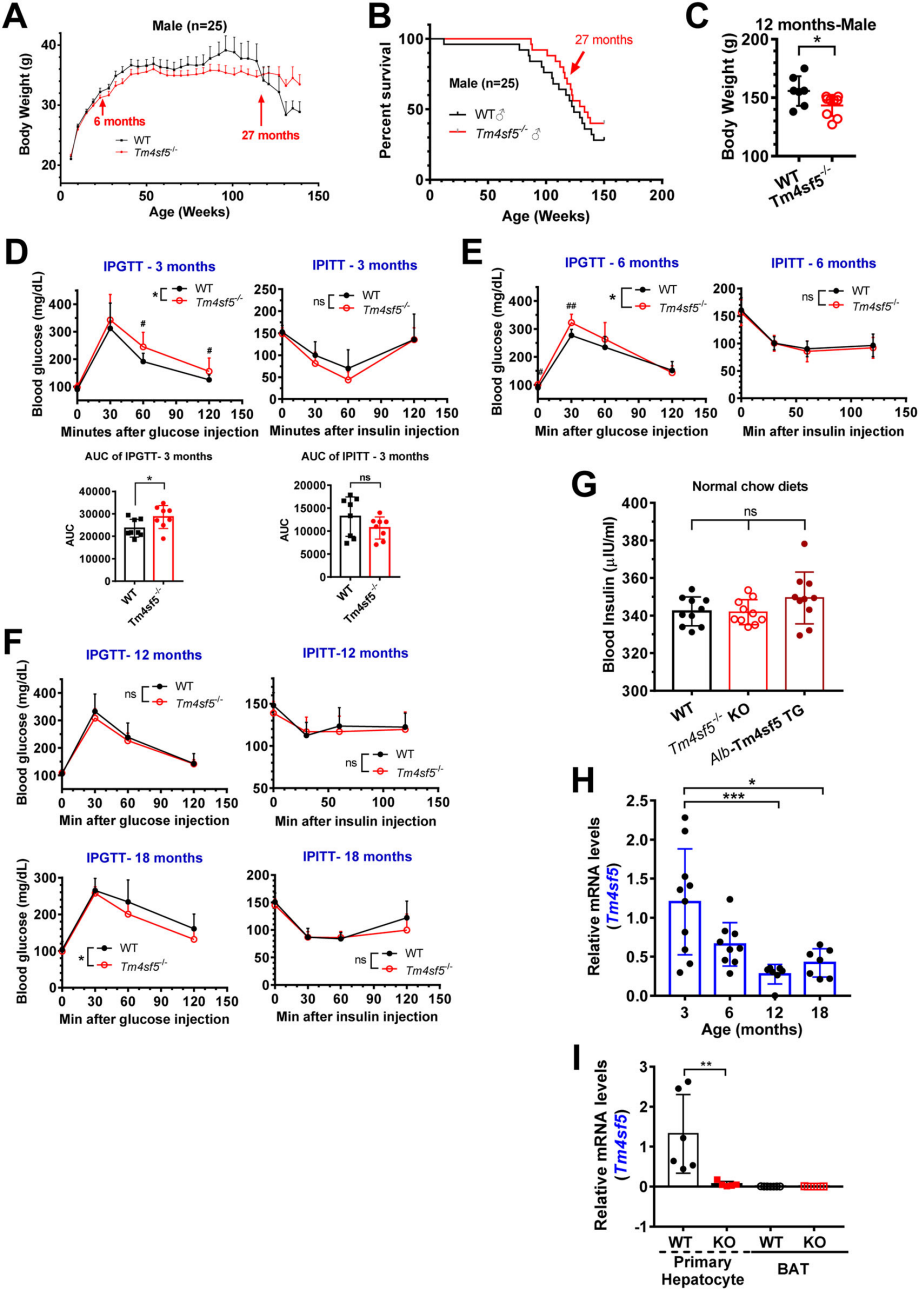
TM4SF5 mediates the differential modulation of blood glucose levels and BW depending on age. (A, B) BW changes (A) and survival rates (B) of WT and *Tm4sf5* ^−/−^ homozygote KO mice (*n* = 25). (C) BWs of 12‐month‐old WT or KO mice (*n* = 7 or 9). (D–F) IPGTT (left) or IPITT (right) using 3‐ (D), 6‐ (E), 12‐ or 18‐month‐old (F) WT or *Tm4sf5* ^−/−^ KO mice (*n* = 9). Data are shown as the mean ± standard deviation (SD). *P < 0.05. ^#^ *P <* 0.05 or ^##^ *P* < 0.01 depicts a statistically significant difference in blood glucose levels between the animal groups at 0 or 30 min, respectively. * indicates *P* < 0.05 for the statistical comparison between WT and KO mice based on the AUC calculations for 0–120 min, whereas ns depicts no significance. (G) Serum insulin levels of 3‐month‐old WT, KO or *Alb*‐Tm4sf5 TG mice (*n* = 10). ns, no significance. (H) Liver tissues from WT mice at the indicated ages were processed to determine *Tm4sf5* mRNA levels. (I) The primary hepatocytes or BAT from 3‐month‐old WT or KO mice were analysed for *Tm4sf5* mRNA. See also Figure S1.

Based on previous reports of TM4SF5‐mediated liver diseases (Kang et al., 2014; Lee et al., 2008; Ryu et al., 2021), we investigated whether TM4SF5 expression could elicit biological effects in animals. For more than 2 years, we have measured BWs and survival rates using age‐matched WT and homozygote *Tm4sf5* KO mice fed a NCD (*n* = 25, Figure 1A,B); we found that 6–27‐month‐old KO mice had lower BWs and greater survival rates compared to WT mice. Interestingly, after 27‐month‐old ages, KO mice maintained BWs rather higher than WT mice (Figure 1A). In another cohort, 12‐month‐old KO mice (*n* = 7–9) had significantly lower BWs than WT mice (Figure 1C). Therefore, we wondered whether the regulation of blood glucose levels involved TM4SF5‐mediated effects, so we measured blood glucose levels following the intraperitoneal injection of glucose or insulin (IPGTT or IPITT, respectively) at different time points. Both 3‐ and 6‐month‐old KO mice were glucose intolerant compared with WT mice (Figure 1D,E). At 12 or 18 months of age, however, KO mice exhibited glucose tolerance without any significant impact by insulin compared with WT mice (Figure 1F). Additionally, as insulin resistance did not appear to be related to glucose (in)tolerance in the KO animals at different ages (Figure 1D–F), 3‐month‐old WT, KO and *Alb*‐Tm4sf5 transgenic (TG) mice (hepatocyte‐specific Tm4sf5 overexpression) showed no significant differences in blood insulin levels when fed an NCD (Figure 1G). Interestingly, *Tm4sf5* mRNA levels were higher at 3‐ and 6‐month‐old‐WT mice (without any significant change) and significantly declined later (Figure 1H), indicating that the TM4SF5 levels might be involved in the regulation of blood glucose levels. Furthermore, primary hepatocytes from WT but not KO mice at 3‐month‐old age expressed *Tm4sf5* mRNA. However, BAT from WT and KO mice did not express *Tm4sf5* mRNA, as reported previously (Choi et al., 2021; Kim et al., 2021) (Figure 1I).

**FIGURE 2:**
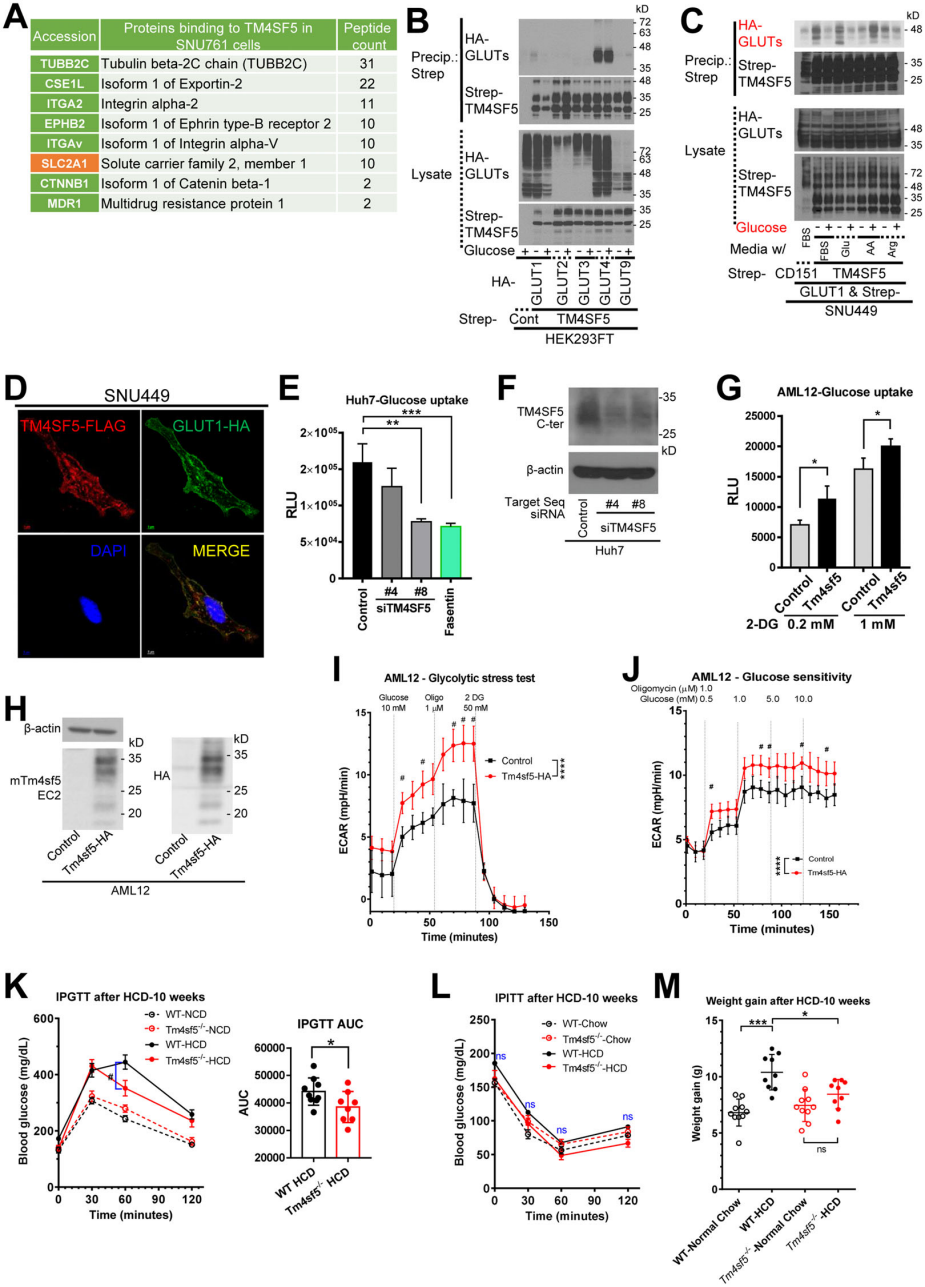
*Tm4sf5^−/−^* KO mice fed an HCD are glucose intolerant irrespective of insulin resistance. (A) Analysis of TM4SF5‐binding proteins in hepatocarcinoma SNU761 cells ectopically expressing TM4SF5‐Strep. (B, C) Immunoblot of HEK293FT (B) and SNU449 (C) for the indicated molecules. Because TM4SF5 can be multimerized (Lee et al., 2006) and palmitoylated or *N*‐glycosylated (Kim et al., 2017), the immunoblots showed blurry bands at 20∼35 kD and multimers at higher molecular weights. (D) Confocal fluorescence images of SNU449 cells stably transfected with TM4SF5‐FLAG stained for TM4SF5‐FLAG (red), GLUT1‐HA (green) and DAPI (blue). (E–H) 2‐DG uptake and TM4SF5 expression in TM4SF5‐suppressed Huh7 cells (E, F) and Tm4sf5‐overexpressing AML12 cells (G, H). *, ** and *** indicate *P* < 0.05, *P* < 0.01 and *P* < 0.001, respectively. (I, J) Glycolytic stress test (I) and glucose sensitivity assay (J) of normal AML12 hepatocytes. ****P < 0.0001. ^#^ *P* < 0.05 for the ECAR values significantly different between control and Tm4sf5‐HA‐psotive AML12 cells at the indicated times (min). Data shown represent three independent experiments. (K–M) IPGTT (K) and IPITT (L) in WT and KO mice (*n* = 10) fed an HCD for 10 weeks. Changes in BW were calculated (M). ns, no significance. *, **, *** and **** for *P* < 0.05, *P* < 0.01, *P* < 0.001 and *P* < 0.0001, respectively. ^#^ depicts for statistically significant difference (*P <* 0.05) for *Y*‐axis values between the sample groups at the indicated time point. See also Figure S2.

Furthermore, when we examined proteins that bound to TM4SF5 via the analysis of TM4SF5 precipitates, we found that different membrane proteins, including GLUT1(SLC2A1), bound to TM4SF5 in hepatocytes (Figure 2A). We further found that GLUT1, GLUT4 and GLUT9 bound to TM4SF5 using coimmunoprecipitation in HEK293FT cells (Figure 2B); GLUT1 binding to TM4SF5 was lost after glucose repletion, whereas GLUT9 binding was increased and GLUT4 binding to TM4SF5 was stable. Further, FBS or glucose repletion led to loss of TM4SF5 binding to GLUT1 in hepatocytes, whereas amino acid repletion stimulated binding, but arginine repletion led to no significant changes in binding (Figure 2C). Thus, the binding of TM4SF5 to GLUT1 (Figure 2D) appeared to be modulated by extracellular glucose levels. Further, TM4SF5 suppression or overexpression reduced or increased glucose uptake in Huh7 HCC (Figure 2E,F) or AML12 murine normal hepatocytes (Figure 2G,H), respectively. Upon treatment with fasentin, a specific inhibitor of GLUT1 and GLUT4 (Wood et al., 2008), glucose uptake into Huh7 cells was also blocked (Figure 2E).

Further, TM4SF5 expression in HEK293A and HEK293T cells resulted in enhanced S6K1 phosphorylation that was dependent on rapamycin‐sensitive mTOR activity, leading to lower ACC phosphorylation to support a favoured energy status upon glucose repletion following glucose and serum starvation (Figure S1A–C). In addition, TM4SF5 overexpression, even in hepatocytes, caused the same effects on S6K1 phosphorylation, lowering the phosphorylation of AMPKα (Figure S1D,E). Such hepatic TM4SF5‐dependent S6K1 activation was abolished by dialysis in serum with a molecular weight (MW) cut‐off of 3 kD (kilodalton) to remove glucose and other compounds and was recovered by the addition of glucose (Figure S1F).

Based on glycolytic stress analyses using measurements of the ECAR, HA‐Tm4sf5 overexpression in AML12 cells (AML12‐HA‐Tm4sf5) resulted in greater glycolytic function than empty control vector (Cont)‐transfected cells (Figure 2I). The ECAR of AML12‐HA‐Tm4sf5 cells was also greatly sensitive to low extracellular glucose concentrations compared to that in AML12‐Cont cells (Figure 2J). ATP production via glycolysis was greater in AML12‐HA‐Tm4sf5 cells compared to AML12‐Cont cells (Figure S2A,B). In contrast, TM4SF5 suppression of Huh7 cells resulted in a lower ECAR, reduced glycolytic ATP production, and an increased ATP production ratio (mitochondrial ATP production/glycolytic ATP production; ATP rate index) (Figure S2C–G).

To examine TM4SF5‐mediated effects in animals fed a HCD (70% kcal carbohydrates), WT or KO mice were analysed following the feeding of an NCD or HCD for 10 weeks. Compared to being fed an NCD, WT and KO mice exhibited glucose intolerance after being fed an HCD. WT mice were more glucose tolerant than KO mice, fed NCD (Figures 1D and 2K); following HCD, KO mice showed better glucose tolerance than WT mice (Figure 2K). This TM4SF5‐mediated improved blood glucose tolerance in animals fed HCD might contradict the observations in cases of NCD and greater glucose uptake in TM4SF5‐positive WT animal (Figures 1D and 2K) or cells (Figure 2E). However, the discrepancy may be due to the difference between chronic NCD and HCD (or abnormal diet); liver tissues of the 3‐month‐old WT animals fed NCD are insignificantly inflammatory (Lee et al., 2022), whereas animals fed HFD or HCD show significant hepatic inflammation for non‐alcoholic steatohepatitis (Lee et al., 2022; Pompili et al., 2020). Meanwhile, compared with WT or KO mice fed an NCD, WT or KO mice fed an HCD showed no significant changes in insulin resistance (Figure 2L). Indeed, when considering the different levels of blood glucose levels at time 0 before the IPITT, the extent of insulin resistance of WT and KO mice fed an HCD was not significantly different. Thus, the glucose intolerance of mice fed an HCD could not be supported by their extent of insulin resistance. Concomitantly, WT mice had significantly enhanced BWs when fed an HCD, unlike KO mice that showed no significant changes in BW (Figure 2M). Therefore, these observations suggest that control of blood glucose levels in mice might be achieved independently of the impact of insulin and that TM4SF5 appears to be involved in, at least kinetically, the regulation of blood glucose levels.

### 3.2 Hepatocyte‐derived sEVs (hep‐sEV^Tm4sf5^) recruited TM4SF5

**FIGURE 3:**
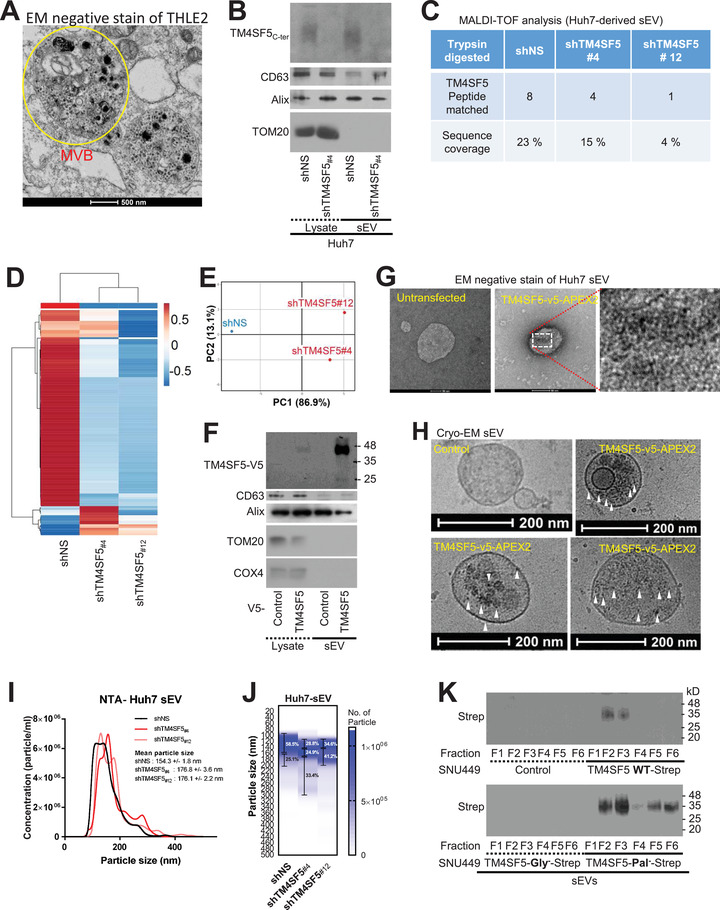
TM4SF5 is recruited into hepatic sEVs. (A) TEM image of Huh7 cells transfected with TM4SF5‐APEX2. Multi‐vesicular body, MVB. (B–E) Western blot analysis for sEV markers and control (B), MALDI‐TOF peptide peak sequence alignment (C), heatmap of common peptide peak intensity profiles (D), and PCA (E) of sEVs from control and TM4SF5‐suppressed Huh7 cells. (F–H) Western blots for v5, sEV markers and controls (F), negative‐stain TEM images (G) and cryo‐EM (H) of sEVs from Huh7 cells transfected with TM4SF5‐APEX2. (I, J) Histogram (I) and heatmap with particle diameters of certain ranges in percentages (J) of sEVs from Huh7 cells stably transfected with shNS or shTM4SF5. (K) SNU449 cells expressing control vector (Cont), Strep‐tagged TM4SF5 WT, *N*‐glycosylation‐deficient (N138A/N155Q, Gly^−^) mutant or palmitoylation‐deficient (C2/6/9/74/75/79/80/84/189A, Pal^−^) mutant were used for sEVs and blotted with Strep‐MAB‐classic‐HRP antibody. The data shown represent three isolated experiments. See also Figure S3

It is reasonable to speculate that a molecular alternative to insulin may be available in TM4SF5‐positive hepatocytes. We thus rationalized that sEVs derived from hepatocytes may play a role in the regulation of blood glucose levels since TM4SF5 metabolically localizes to the lysosomes of hepatocytes (Jung et al., 2019), and tetraspanins, such as TM4SF5, are components of sEVs or exosomes (Gurung et al., 2021). Normal human liver epithelial THLE2 cells transfected with TM4SF5‐APEX2 were visualized using TEM, and APEX2‐mediated dark spots of TM4SF5 localization were observed. TM4SF5‐APEX2 was observed within MVBs (Figure 3A). Further, purification of sEVs from Huh7 cells expressing endogenous TM4SF5 resulted in the release of sEVs containing TM4SF5 (sEV^TM^ ^4SF5^), which was abolished by the cellular transfection of shTM4SF5_#4_, but not a translocase of outer membrane 20 (TOM20) of mitochondria (Figure 3B). In addition, a MALDI‐TOF analysis of sEVs from Huh7 cells showed evidence of TM4SF5‐derived peptides upon digestion with trypsin (Figure 3C) and chymotrypsin (data not shown). A heatmap of the peaks of common peptides and a principal component analysis (PCA) using sEVs derived from Huh7 or TM4SF5‐suppressed Huh7 cells showed differential peptide/protein profiles in the sEVs with or without TM4SF5 expression (Figure 3D,E). Interestingly, the sEVs from TM4SF5‐positive Huh7 cells (transfected with shNS) showed additional protein groups involved in biological adhesion, such as ITGB3, FAM49B, ICAM2, PKP1, SIRPG and ITGBL1, compared to sEVs from TM4SF5‐suppresssd cells (Figure S3A). In addition, tetraspanins including TM4SF5 (but not TM4SF18 or CD151) were found to bind to GLUT4 (Figure S3B).

Furthermore, sEVs from Huh7 cells transfected with or without TM4SF5‐v5‐APEX2 were immunoblotted for the v5 tag and mitochondrial outer (TOM20) or inner membrane protein (cytochrome c oxidase subunit 4, COX4) (Figure 3F). When stained for the v5‐APEX2 signal, we found that TM4SF5‐v5‐APEX2 transfected cells showed dark signals surrounding the sEVs compared to untransfected cells (Figure 3G). Moreover, the sEVs were imaged using cryo‐EM, where 3,3′‐diaminobenzidine staining for the v5 tag was performed 24 h prior to TEM imaging; unlike sEVs from control vector (Cont)‐transfected Huh7 cells, sEVs from TM4SF5‐v5‐APEX2 transfected cells showed signals positive for TM4SF5‐v5‐APEX2 (Figure 3H). To understand the characteristics of sEVs from TM4SF5‐positive or ‐suppressed hepatocytes, their size distributions were analysed by nanoparticle tracking analysis (NTA), resulting in larger sEVs upon TM4SF5 suppression (Figure 3I,J). In addition, sEV fractions were collected following sucrose gradient ultracentrifugation from SNU449 cells stably transfected with Strep control vector, Strep‐tagged TM4SF5‐WT DNA, *N‐*glycosylation‐deficient mutant DNA (N138A/N155Q, Gly^−^) or palmitoylation‐deficient (Pal^−^) mutant DNA prior to immunoblotting. Interestingly, TM4SF5‐WT sEVs were collected in a single peak fraction, whereas TM4SF5‐Pal^−^ sEVs were collected in two peak fractions, and TM4SF5‐Gly^−^ sEVs were not collected in any fractions, indicating the significance of post‐translational modifications of TM4SF5 during its recruitment into sEVs (Figure 3K). Thus, sEV^TM^ ^4SF5^ might have different characteristics than TM4SF5‐negative sEVs in terms of their size and protein profiles.

### 3.3 Glucose repletion causes sEV^TM4SF5^ release from hepatocytes (hep‐sEV^TM4SF5^)

**FIGURE 4:**
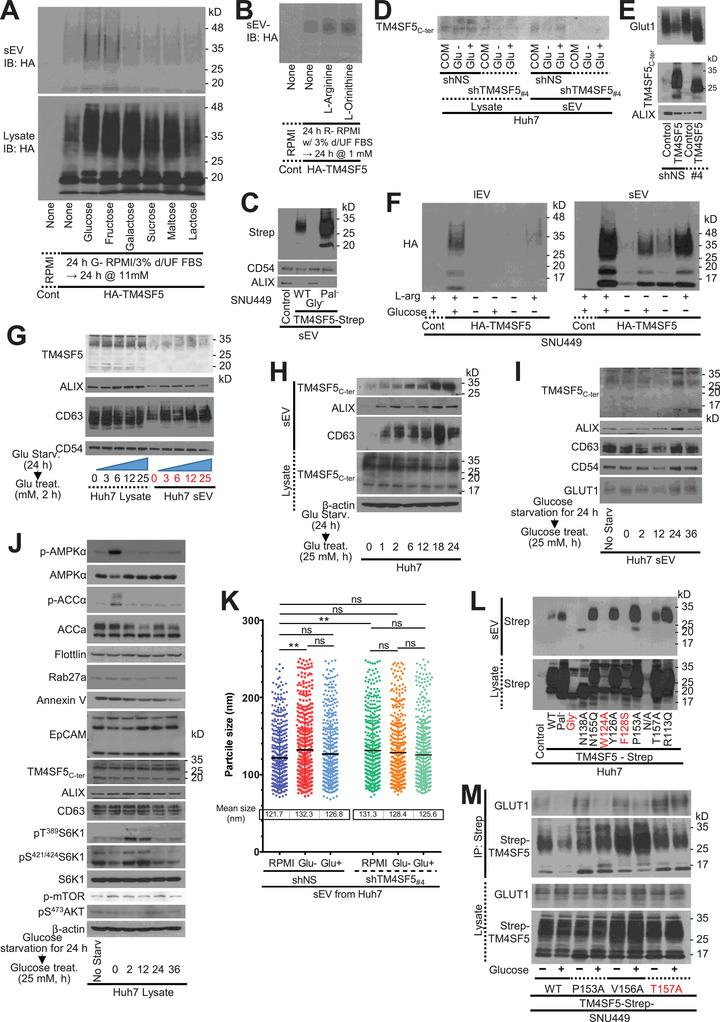
Extracellular glucose causes secretion of TM4SF5‐loaded sEVs from hepatocytes. (A, B) sEVs from cells expressing control HA vector (Cont) or HA‐TM4SF5 were immunoblotted for HA. (C–E) sEVs from cells stably infected or transfected for control vector, indicated cDNAs, or shRNAs were immunoblotted. (F) Induced extracellular vesicles (IEVs) or sEVs from SNU449 cells stably expressing control vector (Cont) or HA‐TM4SF5 were immunoblotted for HA. (G–K) sEVs (G–I and K) or whole‐cell lysates (J) from control Huh7 cells and cells stably transfected with shNS or shTM4SF5_#4_ (K) were immunoblotted (G–J) or subjected to NTA analysis (K). **P < 0.01. ns depicts no significance. (L, M) Whole‐cell lysates or sEVs from Huh7 cells transfected with diverse plasmids for empty control vector (Cont), WT, or mutant TM4SF5‐Strep forms were immunoblotted. Data shown present three independent experiments. See also Figure S4.

Next, we investigated the physiological significance of hep‐sEV^Tm4sf5^. Before immunoblotting to examine TM4SF5 loading, sEVs were collected from TM4SF5‐negative and ‐positive SNU449 cells to which different monosaccharides, disaccharides or amino acids had been added extracellularly in the presence of glucose‐free (G‐) or L‐arginine‐free (R‐) media and 3% dialysed (with MW cut of 3 kD) UF FBS. Interestingly, treatment with glucose, fructose, L‐arginine and L‐ornithine led to TM4SF5 loading into sEVs (Figure 4A,B). As expected by the stabilization of TM4SF5‐Pal^−^ (Kim et al., 2017), palmitoylation‐deficient TM4SF5 was more highly enriched in sEVs, compared with TM4SF5 WT (Figure 4C). Further, glucose depletion of TM4SF5‐positive cells resulted in no sEV^TM4SF5^, and glucose repletion led to the release of sEV^TM4SF5^, although TM4SF5 suppression abolished these effects (Figure 4D). Interestingly, less GLUT1 and Alix were recruited to the sEVs upon TM4SF5 loading, whereas sEVs from TM4SF5‐supppressed cells contained more GLUT1 and Alix, suggesting that TM4SF5 loading of the sEVs may influence the endosomal sorting or packaging of proteins to the sEVs (Figure 4E). When TM4SF5‐positive cells were treated with either L‐arginine or glucose alone, no TM4SF5‐loaded large EVs were generated, whereas sEVs recruited TM4SF5 under the same conditions (Figure 4F). Following glucose starvation, glucose repletion resulted in sEVs that recruited more TM4SF5 in both time‐ and dose‐dependent manners (Figure 4G–I). During the glucose‐mediated loading of TM4SF5 into sEVs, the phosphorylation of AMPK and ACC decreased and that of mTOR1 and S6K1 increased in TM4SF5‐positive cells (Figure 4J), indicating that sEV^TM4SF5^ secretion exhibits favoured energetics and protein translation upon glucose repletion. Further, glucose depletion resulted in a pool of larger sEVs, while glucose repletion decreased the size of the sEV pool, although TM4SF5 suppression abolished these effects (Figures 4K and S4). Recruitment of TM4SF5 WT or TM4SF5 mutants into the sEVs was also examined; *N*‐glycosylation‐deficient (N138A/N155Q, Gly^−^), W124A and F128S mutants were not recruited into the sEVs (Figure 4L). Interestingly, the single mutation (N138A) of a *N‐*glycosylation residue was insufficient for recruitment, whereas the N155Q mutation was quite sufficient for recruitment (Figure 4L). Additional point mutations around N155 allowed recruitment to sEVs, and binding to GLUT1 on the sEVs was still responsive to extracellular glucose, except for the T157A mutation, which was less efficiently recruited compared with the other mutants (Figure 4L,M). These mutant analyses indicate that TM4SF5 could be loaded into sEVs in response to glucose in a specific manner, depending on its structure.

### 3.4 Characteristics of liver/hepatocyte‐derived sEVs depend on TM4SF5 expression

**FIGURE 5:**
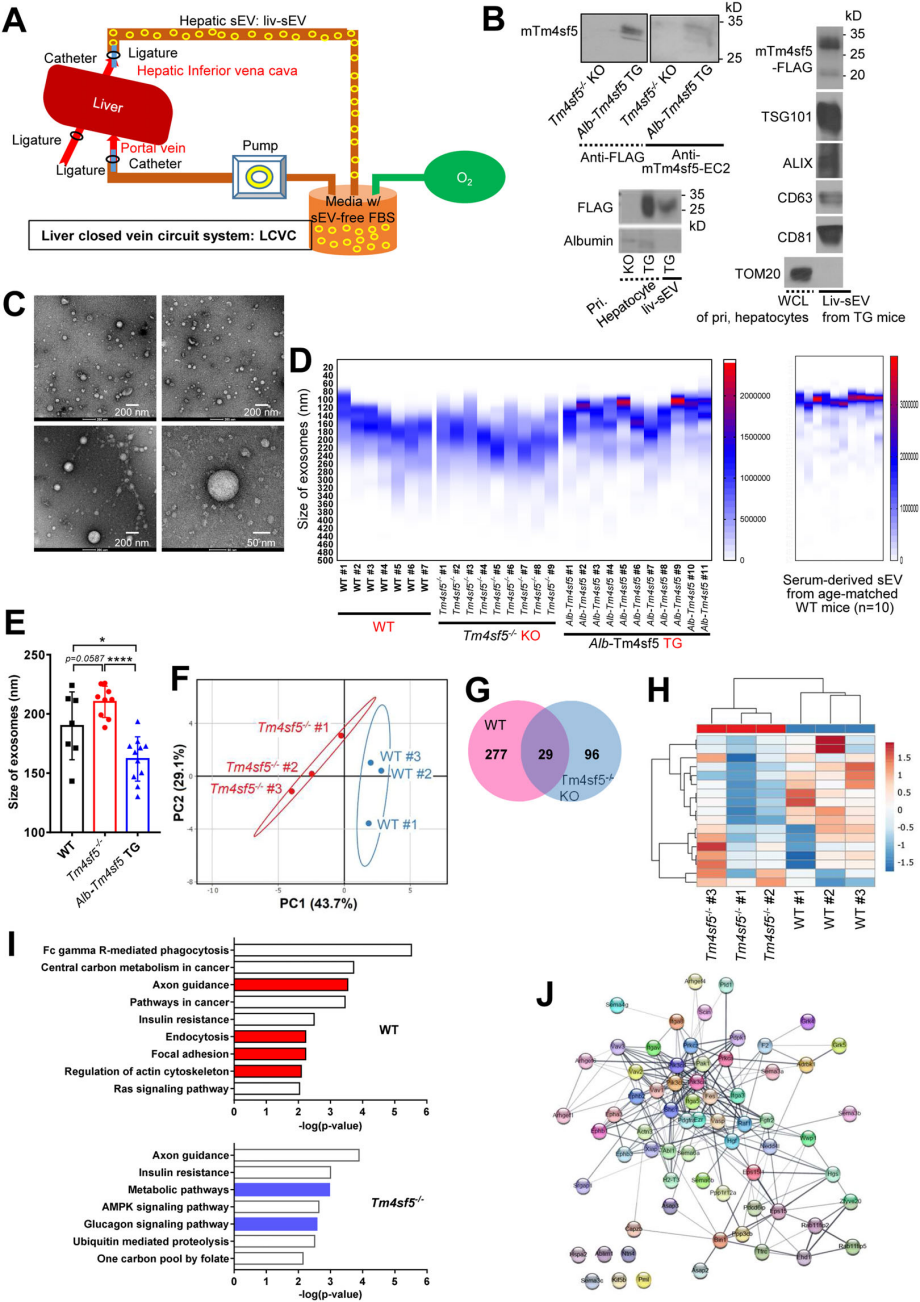
Characteristics of TM4SF5‐loaded sEVs derived from mouse liver or hepatocytes. (A) Schematic diagram of an LCVC system. (B) Liv‐sEVs from *Tm4sf5* ^−/−^ KO or *Alb‐*Tm4sf5 TG C57BL/6N mice or sEVs from primary hepatocytes of KO or TG mice were immunoblotted for the indicated mTm4sf5, FLAG tag and other exosomal markers. (C) Negative‐stain TEM images of liv‐sEVs isolated from LCVC biofluids. (D and E) Size distribution (mean ± SD), heatmap (D) and graphic presentation (E) of liv‐sEVs (D, left and E) from WT, KO and TG mice (*n* = 7∼11) or serum‐derived sEVs (D, right) from 6‐month‐old WT mice. * and **** depict for *P* < 0.05 and *P* < 0.0001, respectively. (F–J) PCA (F), Venn diagram (G), heatmap (H), MALDI‐TOF peptide fingerprinting (I) and protein–protein interactions (J) of liver‐derived sEVs from WT and *Tm4sf5^−/−^* mice (*n* = 3). Circles represent genes, and rectangles represent ontology terms.

While examining the physiological significance of sEVs originating from hepatocytes in vivo, one should consider exosomes or sEVs only from the liver and not from the bloodstream. Thus, we used a liver‐closed vein circuit (LCVC) system, where blood is collected only from intrahepatic circuits via ligatures around the portal vein and inferior vena cava prior to sEV preparation (Figure 5A). The mTm4sf5 protein from *Alb‐*Tm4sf5‐FLAG TG mice was recruited to LCVC‐mediated sEVs (or liv/hep‐sEV^Tm4sf5‐FLAG^) from TG mice together with other sEV markers, including TSG101, ALIX, CD63 and CD81 but not TOM20 (Figure 5B). The liv/hep‐sEV^Tm4sf5‐FLAG^ were observed by electron microscopy to be smaller than 250 nm in diameter (Figure 5C). Further, as shown in sEV^Tm4sf5^ from hepatocytes, the mean size of liv‐sEVs from WT mouse livers was smaller than that from KO mouse livers. Additionally, liv/hep‐sEV^Tm4sf5‐FLAG^ from hepatocyte‐specific Tm4sf5 TG mice (*Alb*‐Tm4sf5‐FLAG TG) comprised a smaller sEV pool than those from WT or KO mouse livers (Figure 5D,E). Meanwhile, sEVs prepared from WT mouse sera were smaller than liv‐sEVs, indicating that sEVs from diverse mouse organs could be smaller than liv‐sEVs (Figure 5D, right). Using label‐free quantification data, we explored the proteomic profiles of sEVs from WT and KO mouse cohorts by employing a PCA, which resulted in a good clustering of the cohorts. The sEVs from WT mice did not overlap with the overall proteomic profile of sEVs from KO mice (Figure 5F). Twenty‐nine proteins were found to be shared in sEVs from WT and KO mouse livers (Figure 5G), whereas 17 proteins were significantly altered in at least one of each group, as shown in a heatmap of common peptide peak intensities. A dendrogram on the left side of the heatmap shows the proteins that clustered together based on their expression profiles in each group of mice (Figure 5H). Further, the Kyoto Encyclopedia of Genes and Genomes pathway database was used for an enrichment analysis of proteins identified by MALDI‐TOF in sEVs from WT and KO mice and revealed that WT‐derived sEVs recruited more proteins involved in dynamic membrane contact or phagocytosis/endocytosis processes, whereas KO‐derived sEVs were loaded with molecules involved in metabolic regulation (Figure 5I). Molecules (red‐highlighted at four dynamic membrane‐rearranging and endocytosis processes) in the WT‐derived sEVs were shown to be involved in a well‐mixed process (Figure 5J). Thus, sEVs from WT mice, unlike those from KO mice, might have features to target cells to areas of dynamic membrane rearrangements.

### 3.5 Liv/hep‐sEV^Tm4sf5^ promote blood glucose clearance in BAT

**FIGURE 6:**
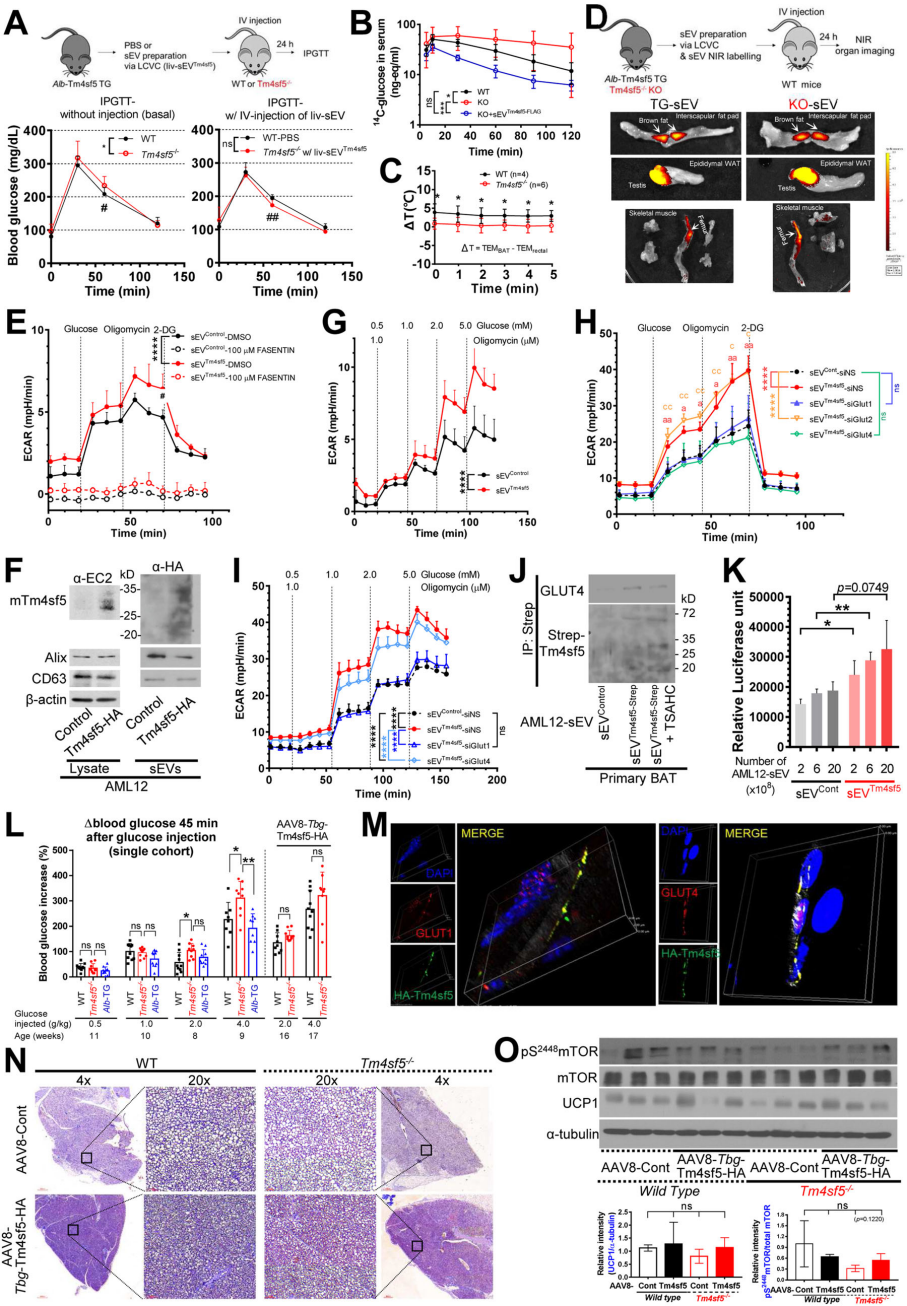
Liv‐sEV^Tm4sf5^ enhance glucose clearance in BAT. (A) IPGTT of WT and *Tm4sf5^−/−^* KO single cohort mice before (left) and after (right) injection of liv‐sEVs from *Alb*‐Tm4sf5 TG mice (*n* = 10). (B) Age‐matched WT and KO male C57BL/6N mice (*n* = 5) without sEV pre‐injection or KO mice (*n* = 6) with a tail vein injection of liv‐sEV^Tm4sf5‐FLAG^ were intraperitoneally injected with ^14^C‐glucose (40 μCi/kg). The blood ^14^C‐glucose levels at various times after glucose injection were assessed using retro‐orbital blood samples for LSC counting. (C) WT or KO mice (*n* = 4 or 6, respectively) were subjected to rectal and interscapular BAT temperature measurement simultaneously at every minute (0∼5 min) following cold exposure for 3 h. *P < 0.05. (D) sEV incorporation into WT mice (*n* = 10) was imaged 24 h after injection of near‐infrared‐dye‐labelled liv‐sEVs from KO or TG mice. (E–G) Glucose stress test (E) and glucose sensitivity assay (G) of primary WT BAT cells treated with either AML12‐sEV^Control^ or AML12‐sEV^HA‐Tm4sf5^ and either DMSO or fasentin. (F) Immunoblots of AML12‐Cont or AML12‐HA‐Tm4sf5 cell extracts and sEVs. (H, I) Glucose stress test (H) and glucose sensitivity assay (I) of primary BAT cells whose various Glut forms were knocked‐down in the presence of AML12‐sEV^Control^ or AML12‐sEV^HA‐Tm4sf5^. (J) AML12‐sEV^Control^ or AML12‐sEV^Tm4sf5‐Strep^ with or without TSAHC (1 μM) pretreatment were exposed to primary WT BAT. sEV protein extracts precipitated using Strep‐agarose beads were immunoblotted using Strep‐MAB‐classic‐HRP and Glut4 antibodies. (K) Glucose uptake assay of primary BAT after treatment with AML12‐sEVs. (L) Blood glucose levels of mice after IP glucose injection. Single cohort [WT, KO and *Alb*‐Tm4sf5 TG (*Alb*‐TG), *n* = 8∼10] mice were used for AAV8‐*Tbg*‐Tm4sf5 injection. (M) Confocal immunofluorescence of BAT cells treated with AML12‐sEV^Tm4sf5‐HA^. (N, O) WT or *Tm4sf5* ^−/−^ KO mice were injected with AAV8‐*Tbg*‐HA‐Tm4sf5, and BAT was analysed by immunohistochemistry for HA/DAB and hematoxylin (N) or by immunoblotting for the indicated molecules (O). (graph) Band intensities normalized with those of α‐tubulin, showing the mean ± SD between groups. * and ** indicate *P* < 0.05 and *P* < 0.01, respectively. ns depicts no significance. ^#^ and ^##^ depict a statistically significant difference (*P <* 0.01 or *P* < 0.01, respectively) for *Y*‐axis values between the sample groups at the indicated time points. Data shown represent three independent experiments. See also Figures S5–S7.

To explore the significance of liv/hep‐sEV^Tm4sf5^ in regulating blood glucose levels, we examined the KO mice intravenously injected with liv/hep‐sEV^Tm4sf5‐FLAG^ from *Alb*‐Tm4sf5‐FLAG TG mice, before comparing them with the parallelly PBS‐injected WT mice. That is, male WT or KO mice (*n* = 10; 10 weeks old) were examined by IPGTT before (i.e. basal) and after injection of PBS or liv/hep‐sEV^Tm4sf5‐FLAG^, respectively. KO mice were more glucose intolerant than WT mice, whereas the KO mice intravenously injected with liv/hep‐sEV^Tm4sf5‐FLAG^ became more glucose‐tolerant than PBS‐injected WT mice (Figure 6A). Interestingly, the basally glucose‐intolerant KO mice became glucose‐tolerant following injection of liv‐sEV^Tm4sf5^ (Figure 6A, red curves). Examination of ^14^C‐labelled glucose intraperitoneally injected into animals resulted in greater clearances of the labelled glucose in WT than KO mice, and liv‐sEV^Tm4sf5‐FLAG^ pre‐injection into the KO mice caused a significant improvement in glucose clearance (Figures 6B and S5). Further, body temperature measurements following cold exposure for 3 h supported interscapular BAT‐based thermogenesis in WT mice greater than KO mice (Figure 6C). TG and KO mouse‐derived sEVs were labelled with the 1,1′‐dioctadecyl‐3,3,3′,3′‐tetramethylindotricarbo‐cyanine iodide near‐infrared fluorescent tracer prior to intravenous injection into WT mice, and 24 h later, various organs were analysed for intravital signals using an in vivo imaging system. The intravenous injection of liv‐sEVs from either TG or KO mice resulted in their incorporation into various organs, including liver, brain, heart, lung, spleen, testis, femur and BAT (Figure 6D), although liv‐sEV^Tm4sf5‐FLAG^ exhibited a greater accumulation in the livers of recipient WT mice (Figures S6A and S6B). The fluorescence of the BAT of recipient WT mice was comparable between TG and KO‐derived liv‐sEV injections (Figure S6C). To clarify the sEV‐mediated effects on the BAT, we performed a glucose stress test and a sensitivity assay using differentiated primary BAT cells treated with sEVs from TM4SF5‐negative normal AML12 murine hepatocytes (AML12‐sEV^Control^) or AML12 cells stably transfected with HA tag‐conjugated mouse Tm4sf5(AML12‐sEV^HA‐Tm4sf5^). Huh7 or AML12 cells were less proliferative in 10% UF‐FBS or d/UF‐FBS than those in 10% normal (i.e. unprocessed glucose‐ and/or sEV‐containing) FBS, suggesting that the sEVs and/or glucose from FBS were depleted, based on the reports that sEVs support cell growth (Eitan et al., 2015; Witwer et al., 2013) (Figure S7). Thus, the application of sEVs prepared from the AML12 hepatocytes without or with Tm4sf5 to in vitro BAT cells could be performed properly for the glucose tolerance or sensitivity tests. ECAR levels of glycolytic capacity were greater in BAT cells treated with AML12‐sEV^HA‐Tm4sf5^ than in cells treated with AML12‐sEV^Control^, whereas cells treated with AML12‐sEVs showed negligible ECAR levels upon treatment with fasentin (Figure 6E), a specific inhibitor of GLUT1 and GLUT4 (Wood et al., 2008). As expected, mouse Tm4sf5 exogenously transfected to normal Tm4sf5‐null AML12 murine hepatocytes could release sEVs loaded with Tm4sf5 (AML12‐sEV^HA‐Tm4sf5^; Figure 6F). Further, AML12‐sEV^HA‐Tm4sf5^ caused differentiated BAT cells to be more sensitive to glucose (Figure 6G). To define the roles of the different Gluts in the AML12‐sEV^HA‐Tm4sf5^‐mediated effects on the glycolytic capacity of BAT cells, differentiated BAT cells were first transfected with either siRNA against Glut1, Glut2 or Glut4 prior to the AML12‐sEV treatment. Glycolytic capacity was enhanced in differentiated primary BAT cells by AML12‐sEV^HA‐Tm4sf5^ compared with AML12‐sEV^Control^; these effects were abolished by suppression of Glut1 or Glut4, but not of Glut2 (Figure 6H). These observations suggest that the glycolytic capacity of BAT is moderated by AML12‐sEV^HA‐Tm4sf5^ targeting and involves Glut1 and Glut4. Furthermore, the glucose sensitivity of BAT cells was analysed following treatment with either sEV^Control^ or sEV^HA‐Tm4sf5^ from AML12 cells in which Glut1 or Glut4 had been suppressed. Interestingly, sEVs from AML12 cells after Glut1 (but not Glut4) suppression abolished the glucose sensitivity of BAT cells that had been stimulated by AML12‐sEV^HA‐Tm4sf5^, indicating that Glut1 in Tm4sf5‐positive hepatocytes is important for the AML12‐sEV^HA‐Tm4sf5^‐mediated effects on BAT glycolysis (Figure 6I). Further, sEVs from AML12 cells stably transfected with control vector (Cont) or Tm4sf5‐Strep vector were treated to the differentiated primary BAT cells prior to coimmunoprecipitation of Tm4sf5‐Strep and Glut4. Interestingly, BAT cells treated with AML12‐sEV^Tm4sf5‐Strep^, but not with AML12‐sEV^Cont^, showed coprecipitation of Tm4sf5‐Strep with Glut4, which was reduced by pre‐treatment of AML12‐sEV^Tm4sf5‐Strep^ with TSAHC [a specific TM4SF5 inhibitor (Lee et al., 2009)] (Figure 6J), indicating that AML12‐sEV^Tm4sf5^ can associate with Glut4 during sEV targeting. Analysis of glucose uptake into differentiated BAT cells following treatment with different concentrations of AML12‐sEV^HA‐Tm4sf5^ showed that glucose uptake is favoured by sEV^HA‐Tm4sf5^ compared with sEV^Control^ (Figure 6K).

Based on observations showing that sEV^Tm4sf5^ acts on BAT, we wondered whether this finding was the result of wasting excess glucose that liver, muscle and white adipose tissues are unable to metabolize. To evaluate this hypothesis, we performed a series of GTTs by injecting glucose at increasing doses into age‐matched WT, KO and TG mice (*n* = 9–10 at 3 months of age) to see how *Tm4sf5*‐engineered animals manage blood glucose levels. Single cohort animals were injected with glucose at doses of 0.5, 1.0, 2.0 or 4.0 g/kg each week, and blood glucose levels were measured 45 min later. Interestingly, when lower doses of glucose (e.g. 0.5 and 1.0 g/kg) were injected, the animals showed no significant differences in their blood glucose levels, independent of Tm4sf5 expression, whereas KO mice showed significantly higher blood glucose levels than WT or TG mice when higher (2.0 and 4.0 g/kg) doses of glucose were injected (Figure 6L). To determine whether TM4SF5 was indeed responsible for the differences observed, we injected AAV8‐*Tbg*‐HA‐Tm4sf5 virus [adeno‐associated virus subtype 8(AAV8) with the hepatocyte‐directed thyroxine‐binding globulin (*Tbg*) promoter and HA‐tagged *Tm4sf5* gene (Wei et al., 2021)], into WT or KO mice in the same cohort 1 day after the last GTT. Five to six weeks later, another GTT was performed, and blood glucose levels were determined 45 min after an injection of 2.0 or 4.0 g/kg glucose. AAV8‐mediated expression of Tm4sf5 in KO mice resulted in no significant difference in glucose levels, even after high extracellular glucose treatment (Figure 6L). These observations support that TM4SF5 expression plays a role in the modulation of blood glucose levels.

Next, we evaluated whether AML12‐sEV^HA‐Tm4sf5^ could enter differentiated BAT cells using confocal immunofluorescence with reconstruction of 41 Z‐Stack slices with a 0.1‐μm thickness. AML12‐sEV^HA‐Tm4sf5^ were colocalized with GLUT1 or GLUT4 on the cell surface and perinuclear area of BAT cells (Figure 6M). Immunohistochemistry results of BAT from WT or KO mice injected with AAV8‐Cont or AAV8‐*Tbg*‐HA‐Tm4sf5 virus showed HA‐Tm4sf5 positive signals in the BAT of WT and KO mice 3 weeks after the injections; additionally, AAV8‐*Tbg*‐HA‐Tm4sf5 virus injection resulted in fewer fat droplets, presumably supporting improved thermogenesis (Figure 6N). Since BAT utilizes glucose to dissipate energy as heat at a high rate, mainly via the function of uncoupling protein 1 (UCP1) to uncouple the mitochondrial proton gradient from ATP synthesis (Tabuchi & Sul, 2021), we wondered whether the effects of the AAV8‐*Tbg*‐HA‐Tm4sf5 virus on BAT might be related to the expression of UCP1. Interestingly, we did not observe any significant differences in UCP1 expression in the BAT of animals injected with either AAV8‐EV or AAV8‐*Tbg*‐HA‐Tm4sf5 (Figure 6O). Meanwhile, as the movement of glucose into BAT is shown to be dependent on the mTor pathway (Olsen et al., 2017), we found that the phosphorylation of mTOR was increased (insignificantly at *P* = 0.1220) in KO (but not WT) mice injected with the AAV8‐*Tbg*‐HA‐Tm4sf5 virus, compared to that of KO mice injected with the AAV8‐Cont virus (Figure 6O). These observations suggest that hepatocyte Tm4sf5 might play a role in BAT‐mediated clearance of excess blood glucose, presumably leading to eventual thermogenesis of BAT, as shown in FIgure 6C.

## 4 DISCUSSION

We showed evidence in this study that hepatocyte TM4SF5 is involved in the regulation of glucose metabolism and homeostasis with clearance of excess blood glucose as heat in BAT. In the liver, TM4SF5 expression led to enhanced glucose uptake, activation of mTOR/S6K1 signalling and efficient energy status via glycolytic ATP production. Interestingly, extracellular glucose repletion following depletion caused secretion of TM4SF5‐loaded sEVs from hepatocytes, although TM4SF5‐negative sEVs were still generated under conditions of glucose starvation. These hepatocyte‐ or liver‐derived sEVs can traverse to other organs for metabolic purposes. TM4SF5 in HEK293FT cells bound to GLUT1 and GLUT4, suggesting that TM4SF5 might play a role in adipose tissue and/or muscle expressing GLUT4. These organs are major sites of excess blood glucose disposal via storage or energy generation (Pozo & Claret, 2018). Indeed, extracellular glucose stimulation of TM4SF5‐loaded sEVs (sEV^Tm4sf5^) showed characteristics different from sEV^Control^ lacking TM4SF5; sEV^Tm4sf5^ were smaller in diameter than sEV^Control^ and exhibited different protein profiles, including cell–cell adhesion‐related molecules, such as ITGB3, FAM49B, ICAM2, PKP1, SIRPG and ITGBL1. We also purified sEVs via the LCVC system (i.e. liv‐sEV^Tm4sf5^). Such liv/hep‐sEV^Tm4sf5‐FLAG^ were shown to target BAT by intravital imaging after intravenous injection into WT mice. Both liv/hep‐sEV^Control‐FLAG^ (or KO‐sEVs) and liv/hep‐sEV^Tm4sf5‐FLAG^ (or TG‐sEVs) were incorporated into BAT at comparable levels. Treatment of KO mice with liv/hep‐sEV^Tm4sf5‐FLAG^ from the livers of *Alb*‐Tm4sf5 TG mice improved blood glucose tolerance compared with WT mice. sEV^Tm4sf5^ may thus function like insulin, which appears to be supported by the observation that sEV^Tm4sf5^ colocalizes with GLUT4 in BAT. Therefore, TM4SF5 in hepatocytes may modulate homoeostatic blood glucose levels as shown in this study, although hepatic TM4SF5 also play roles in the development of pathological phenotypes for non‐alcoholic fatty liver disease (Lee et al., 2022; Ryu et al., 2021) and hepatocellular carcinoma (Lee et al., 2008; Sun et al., 2021). These chronic liver diseases are associated with the abnormal metabolic and/or inflammatory situations. Therefore, TM4SF5 expression levels depending on the animal age may be involved in regulating blood glucose levels and BWs, the abnormality of which may promote chronic liver diseases. Poor healthy (e.g. sarcopenia or frailty) situations can be linked to chronically abnormal metabolisms (e.g. chronic type 2 diabetes via insulin resistance and inflammation) with lower BWs or body mass indexes (due to lower muscle mass and a lean status) at later ages (Izzo et al., 2021), such as the 110‐week‐old age of mice as shown in this study. The induction of insulin resistance in old rats by sucrose feeding results in significant loss of lean body mass, increased fat mass and reduced muscle protein synthesis, supporting a causal effect of insulin resistance on sarcopenia in the elderly (Gatineau et al., 2015). Thus, the metabolic status and life quality compared with aging may involve TM4SF5‐mediated roles in regulating blood glucose levels via cross‐talk between the liver and BAT.

The significance of TM4SF5 recruitment into liv‐sEVs might involve the regulation of preferred organ tropisms. The size and molecular composition of sEVs from diverse organs differ and guide their biodistribution (Zhang et al., 2018). In addition to smaller diameters, sEV^Tm4sf5^ exhibited different protein profiles, including integrins, FAM49B, ICAM2, PKP1 and SIRPG. Specific integrins on tumour‐derived exosomes or sEVs lead to specific binding to target cells during exosome‐mediated cancer metastasis (Paolillo & Schinelli, 2017), such as integrin αvβ5 for liver Kupffer cells and α6β1/α6β4 for lung fibroblasts and epithelial cells (Hoshino et al., 2015). The integrin α4β7 is also essential for the homing of T‐cell exosomes in the small intestine (Park et al., 2019). The extracellular matrix in specific organs may further specify the target organs for sEVs loaded with integrins as their receptors (Buzas et al., 2018). In addition, ICAMs are involved in cell–cell communication and adhesion (Harjunpaa et al., 2019). Plakophilin‐1 (PKP1) and ICAM1 proteins were found in sEVs purified from serum 4 weeks after surgery in a mouse model of spared nerve injury compared to sham control mice, appearing to play roles in vesicular protein sorting under conditions of neuropathic pain (Jean‐Toussaint et al., 2020). Signal‐regulatory protein gamma (SIRPγ, SIRPG) is an immunoglobulin superfamily ligand expressed in T‐cells and some B‐cells that binds endothelial cell CD47 and plays a role in the remodelling of the actin cytoskeleton during leucocyte transmigration through the endothelium (Azcutia et al., 2012). As a tetraspanin, TM4SF5 might thus function in the packaging of additional protein components for more efficient incorporation of liv‐sEVs to BAT. Interestingly, a previous report reveals an adipose to liver extracellular vesicle communication axis, which can form a counterpart to the liver to adipose axis that this study describes (Thomou et al., 2017).

Excess extracellular glucose levels caused secretion of liv/hep‐sEV^Tm4sf5^ that traversed to the BAT of WT or KO mice, based on intravital imaging for their targeting to BATs of WT or KO mice and immunohistochemical and western blot analyses of BAT after viral application of Tm4sf5 to hepatocyte/livers. Such liv/hep‐sEV^Tm4sf5‐FLAG^ targeting to BAT led to efficient Glut4‐dependent glucose uptake and glycolytic ATP production presumably for thermogenesis, compared with liv/hep‐sEV^Control^. AML12‐sEV^Tm4sf5^ has been shown to bind GLUT4, which can be expressed in BAT in response to insulin (Assimacopoulos‐Jeannet et al., 1991). TM4SF5 binds diverse membrane proteins and receptors to form massive protein–protein complexes (Lee, 2014, 2015), termed TM4SF5‐enriched microdomains (i.e. T_5_ERMs), similar to tetraspanin‐enriched microdomains (Charrin et al., 2009; Yanez‐Mo et al., 2009). TM4SF5 in hepatocytes binds integrins (Choi et al., 2009), EGFR (Kim et al., 2017), CD151(Kang et al., 2014), CD44 (Lee et al., 2015), mTOR (Jung et al., 2019), FAK (Jung et al., 2012), c‐Src (Song et al., 2021) and transporters for nutrients including amino acids (Jung et al., 2019; Kim et al., 2019) and glucose (this study). Based on such TM4SF5 characteristics, the packaging of specific membrane proteins and/or receptors into liv/hep‐sEV^Tm4sf5^ might be achieved via protein–protein associations within T_5_ERMs. Therefore, Tm4sf5 in liv‐sEV^Tm4sf5^ might efficiently target BAT where Glut4 is expressed and respond to insulin for blood glucose clearance (Assimacopoulos‐Jeannet et al., 1991). While sEV^Tm4sf5^ appear to be loaded with less Alix (Figures 3 and 4), they contain similar levels of other exosomal markers. Alix causes the specific accumulation of proteins, including **e**ndosomal‐**s**orting **c**omplexes **r**equired for endosomal **t**ransport (ESCRTs) in the endosomes during exosome biogenesis (Larios et al., 2020). Alix depletion decreases the exosomal loading of tetraspanins CD9, CD81 and CD63, suggesting that a mutual regulation of exosomal markers or proteins might be possible, although protein loading or sorting into exosomes can be regulated by more than one mechanism, and/or specific cells can produce more than one exosomal population (Tkach & Thery, 2016; van Niel et al., 2018). TM4SF5 was previously shown to regulate the expression and intracellular localization of CD63 (Kang et al., 2014). TM4SF5 may thus be involved in sEV‐BAT cell adhesion for metabolic regulation.

TM4SF5 expression appears to be important for glucose metabolism; *Tm4sf5* ^−/−^ KO C57BL/6N male mice showed higher blood glucose levels at 3 or 6 months of age compared to age‐matched WT mice, whereas KO mice older than 12 months of age (i.e. 12 or 18 months) showed the opposite phenotype. Meanwhile, over their life spans, KO and WT mice did not exhibit any significant differences in insulin resistance whereas *Tm4sf5* levels were declined after approximately 6 months of age. Further, the higher glucose intolerance observed in KO mice compared to WT mice at 3 months of age improved after the injection of liv/hep‐sEV^Tm4sf5‐HA^ from *Alb*‐Tm4sf5 TG mice. The effects of liv/hep‐sEV^Tm4sf5^ on targeting to BAT may presumably modulate blood glucose levels and BW differentially, depending on age; at younger ages like 6 months (equivalent to a human age of 30 years (Flurkey et al., 2007; Wang et al., 2020)), hepatic TM4SF5 expression may be correlated with lower blood glucose levels, whereas higher blood glucose levels might be observed at older ages (>12 months of age, which is equivalent to a human age of 40 years). Such a pattern over the life span was correlated with the expression pattern of *Tm4sf5* mRNA, although it would be interesting to know how ageing led to declined *Tm4sf5* expression. As for changes in BW, KO and WT mice showed similar patterns until 4 months of age; thereafter, KO mice exhibited a lower BW than WT mice until 27 months of age (equivalent to a human age of 70 years) and maintained a higher BW compared to WT mice. Indeed, glucose uptake and thermogenesis in BAT upon β3‐adnergic stimulation that is supported by mTOR activity may be relevant to ameliorating obesity and diabetes (Olsen et al., 2017). By maintaining their BW, KO mice might eventually be able to survive longer than WT mice. Thus, excess glucose clearance in BAT stimulated by liv/hep‐sEV^Tm4sf5^ appears relevant to changes in BW and presumably furthers longevity, although additional studies on the TM4SF5‐mediated aspects of these mechanisms are needed.

## AUTHOR CONTRIBUTIONS

Jae Woo Jung designed and performed experiments and wrote the 1st version, in parts, of the Materials and Methods and the Figure legends; Eunmi Kim did revisional experiments; Ji Eon Kim performed WB and IHC with animal tissues; Hyejin Lee, Haesong Lee and Eun‐Ae Shin helped with constructs and reagents; Jung Weon Lee designed experiments, interpreted the data and wrote the manuscript.

## CONFLICT OF INTEREST

The authors declare no potential conflicts of interest.

## Supporting information


**Figure S1. Extracellular glucose promoted TM4SF5‐dependent mTOR and S6K1 activity**. Stable cells expressing control empty vector or TM4SF5 (A, B, C, and F) or cells infected with lentivirus for control shRNA against non‐specific (shNS) or TM4SF5 sequences (#4, #5, or #8 in Table 1, D and E) were glucose‐starved for the indicated periods. Glucose repletion was then done for various times without or with other treatments as indicated, before harvesting whole cell extracts. The whole cell extracts were immunoblotted for the indicated molecules. The data shown are representative for three isolated experiments. See also Figure 1.
**Figure S2. TM4SF5‐positive hepatocytes showed a greater glycolytic activity for ATP production**. Normal murine hepatocyte AML12 stably expressing empty vector (Control) or HA‐TM4SF5 (A and B) or Huh7 hepatocarcinoma cells stably expressing siRNA against control scrambled sequence (siNS) or TM4SF5 sequences (#7 and 8 sequences in Table 1, C‐F) were processed to immunoblots and qRT‐PCR (C), or seahorse analysis (D) for the ATP production rates or for ATP rate index (E to G, mitochondrial ATP production/glycolytic ATP production). Data shown represent three independent experiments. See also Figure 2.
**Figure S3. MALDI‐TOF analysis of sEVs from Huh7 cells without or with TM4SF5 suppression, and TM4SF5 binding to GLUT4. (A)** The sEVs were prepared from TM4SF5‐positive Huh7 cells (transfected with shNS, Top in A) or TM4SF5‐suppressed cells (with transfection of shTM4SF5 (against #4 or #12 sequences in Table 1: A, middle and bottom), before processing to MALDI‐TOF proteomic analysis. Further, the proteins in the TM4SF5‐positive or ‐negative sEVs were processed for PANDER (Protein Analysis Through Evolutionary Relationships, http://pantherdb.org) with Gene Ontology (GO) for biological process (PANDER‐GO‐slim). TM4SF5‐positive sEVs showed additional proteins with red‐highlighted names and circle (A, top). **(B)** HEK293FT cells were transfected with control or different plasmids for the indicated molecules for 48 h. N/A indicates untransfected cell lysates although transfection has been performed. Whole cell lysates were precipitated for Strep‐tagged molecules prior to blotting for HA‐GLUT4. See also Figure 3.
**Figure S4. Sizes in diameters of sEVs from Huh7 hepatocytes were increased by glucose‐starvation, which were reduced by glucose repletion. (A)** sEVs or exosomes (Exo) from Huh7 hepatocytes with glucose depletion (w/o, for 48 h) or 24 h‐repletion (following 24 h‐depletion, w/) were analyzed for their size distribution in particle diameters (nm) by turnable resistive pulse sensing (TRPS). **(B)** Purified huh7 sEVs after size‐exclusion chromatography in complete media (w/ RPMI), glucose‐starved (w/ Glu‐), or glucose‐repleted (w/ Glu+) were analyzed by nanoparticle tracking analysis (NTA). Data shown represent three independent experiments. See also Figure 4.
**Figure S5. Clearance of blood ^14^C‐glucose intraperitoneally injected into the KO mice was improved by liv‐sEV^Tm4sf5‐FLAG^ injection**. Age‐matched WT and KO male C57BL/6N mice (n=5) without any sEV pre‐injection or KO mice (n=6) with a tail‐vein‐injection of liv‐sEV^Tm4sf5‐FLAG^ were intraperitoneally injected with ^14^C‐glucose (40 μCi/kg). At several time points (5, 10, 30, 60, 90, and 120 min) after glucose injection, retro‐orbital blood samples were processed for the LSC counting, to observe clearance of blood ^14^C‐glucose depending on Tm4sf5 expression and liv‐sEV^Tm4sf5‐FLAG^ administration. See also Figure 6.
**Figure S6. In vivo analysis of WT mice intravenously injected with labeled‐liv‐sEVs from either *Tm4sf5* TG or KO mice. (A to C)** Near‐infrared‐dye‐labeled liv‐sEVs purified via the liver‐closed vein circuit (LCVC) using *Tm4sf5^‐/‐^* KO or (*Alb*‐Tm4sf5) TG mice were intravenously injected into the WT mice (n=10). One day later, the signals for sEVs in different organs were measured using intravital in vivo imaging system (A and B). The fluorescent signals for the sEVs incorporated into BAT were compared. See also Figure 6.
**Figure S7. Less supports for hepatocyte proliferation rate by sEVs‐depleted or sEV/glucose‐depleted FBS**. Huh7 or AML12 cells (5 x 10^4^ cells/well) were seeded in either normal 10% FBS‐, UF‐FBS, or d/UF‐FBS and counted again after the indicated times. UF‐FBS was prepared to deplete sEVs in the serum and d/UF‐FBS was to remove the glucose in the UF‐FBS, as explained in the Materials and Methods. The 24‐hour proliferation rate was calculated and the rates of UF‐ and d/UF‐treated cells were normalized to its control (i.e., cultured in normal sEVs‐containing FBS). Statistics comparisons performed by two‐way ANOVA. **, ***, or **** depict *P*<0.01, 0.001, or 0.0001 for statistical significance. See also Figure 6.
