# Supplementary material for: Liver‐originated small extracellular vesicles with TM4SF5 target brown adipose tissue for homeostatic glucose clearance
Source: J Extracell Vesicles. 2022 Sep 5;11(9):e12262. doi: 10.1002/jev2.12262 (PMC9443943; doi:10.1002/jev2.12262)
Supplement: Supplementary file 1 — Figure S1. Extracellular glucose promoted TM4SF5‐dependent mTOR and S6K1 activity. Stable cells expressing control empty vector or TM4SF5 (A, B, C, and F) or cells infected with lentivirus for control shRNA against non‐specific (shNS) or TM4SF5 sequences (#4, #5, or #8 in Table 1, D and E) were glucose‐starved for the indicated periods. Glucose repletion was then done for various times without or with other treatments as indicated, before harvesting whole cell extracts. The whole cell extracts were immunoblotted for the indicated molecules. The data shown are representative for three isolated experiments. See also Figure 1. Figure S2. TM4SF5‐positive hepatocytes showed a greater glycolytic activity for ATP production. Normal murine hepatocyte AML12 stably expressing empty vector (Control) or HA‐TM4SF5 (A and B) or Huh7 hepatocarcinoma cells stably expressing siRNA against control scrambled sequence (siNS) or TM4SF5 sequences (#7 and 8 sequences in Table 1, C‐F) were processed to immunoblots and qRT‐PCR (C), or seahorse analysis (D) for the ATP production rates or for ATP rate index (E to G, mitochondrial ATP production/glycolytic ATP production). Data shown represent three independent experiments. See also Figure 2. Figure S3. MALDI‐TOF analysis of sEVs from Huh7 cells without or with TM4SF5 suppression, and TM4SF5 binding to GLUT4. (A) The sEVs were prepared from TM4SF5‐positive Huh7 cells (transfected with shNS, Top in A) or TM4SF5‐suppressed cells (with transfection of shTM4SF5 (against #4 or #12 sequences in Table 1: A, middle and bottom), before processing to MALDI‐TOF proteomic analysis. Further, the proteins in the TM4SF5‐positive or ‐negative sEVs were processed for PANDER (Protein Analysis Through Evolutionary Relationships, http://pantherdb.org) with Gene Ontology (GO) for biological process (PANDER‐GO‐slim). TM4SF5‐positive sEVs showed additional proteins with red‐highlighted names and circle (A, top). (B) HEK293FT cells were transfected with control or d [file JEV2-11-e12262-s001.docx]

**Liver-originated small extracellular vesicles with TM4SF5 target brown adipose tissue for homeostatic glucose clearance**

Jae Woo Jung^1,2,3,#^, Ji Eon Kim^1,2,#^, Eunmi Kim^1,2,#^, Hyejin Lee^1^, Haesong Lee^1^, Eun-Ae Shin^1^, and Jung Weon Lee^1,2,3,*^.

Supplementary figures

Figure S1


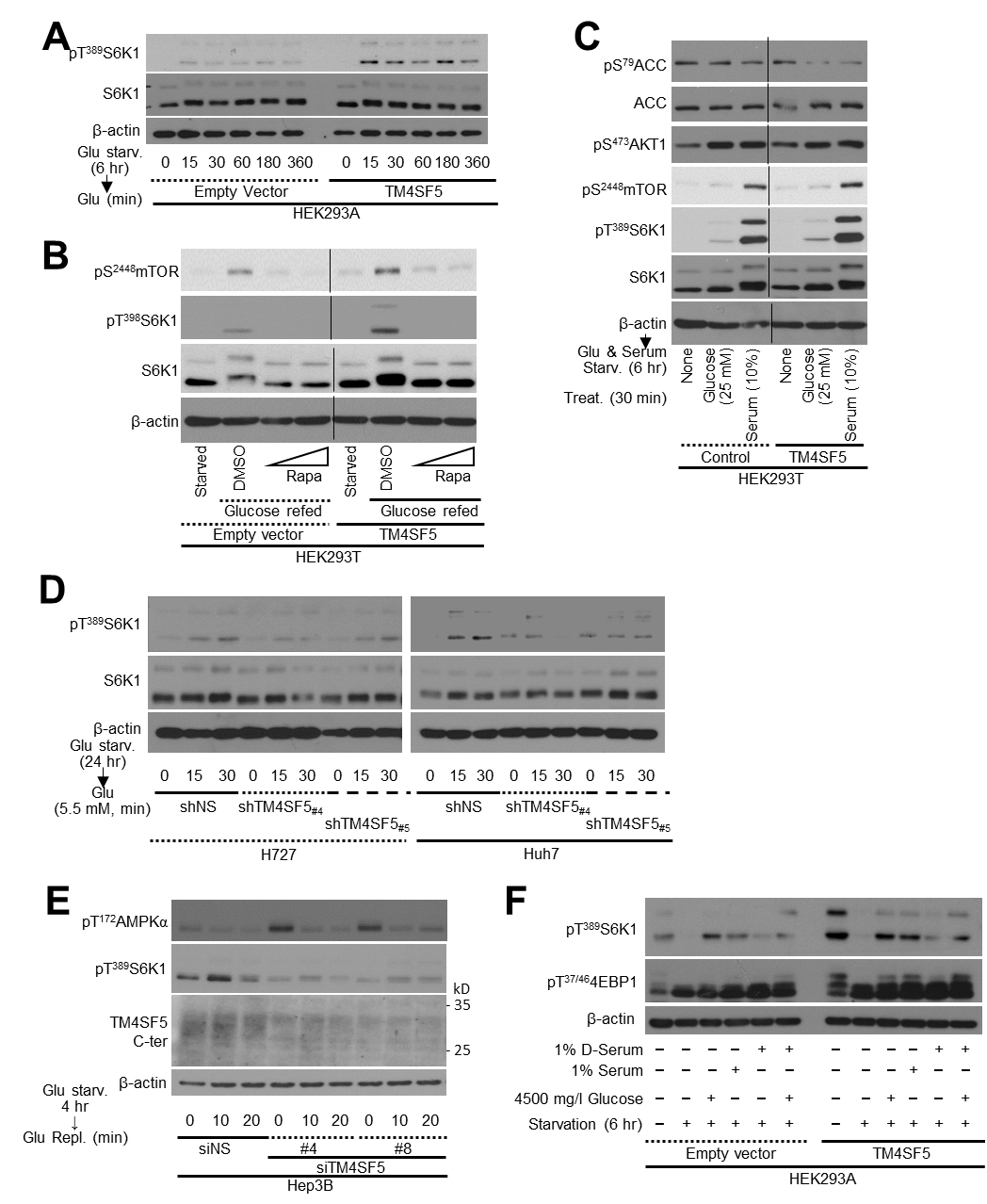


**Figure S1. Extracellular glucose promoted TM4SF5-dependent mTOR and S6K1 activity.** Stable cells expressing control empty vector or TM4SF5 (A, B, C, and F) or cells infected with lentivirus for control shRNA against non-specific (shNS) or TM4SF5 sequences (#4, #5, or #8 in Table 1, D and E) were glucose-starved for the indicated periods. Glucose repletion was then done for various times without or with other treatments as indicated, before harvesting whole cell extracts. The whole cell extracts were immunoblotted for the indicated molecules. The data shown are representative for three isolated experiments. See also Figure 1.

Figure S2.


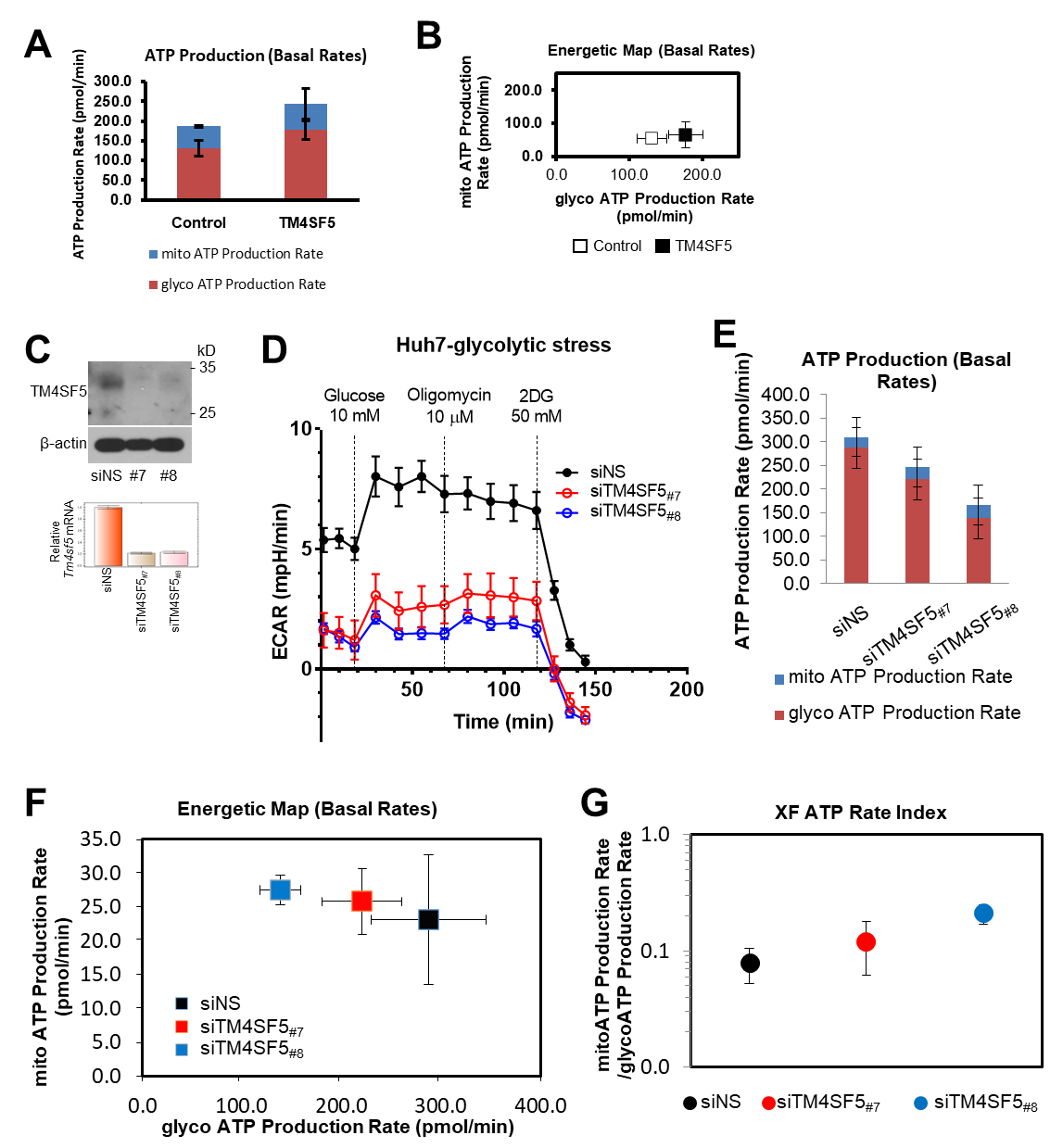


**Figure S2. TM4SF5-positive hepatocytes showed a greater glycolytic activity for ATP production.** Normal murine hepatocyte AML12 stably expressing empty vector (Control) or HA-TM4SF5 (A and B) or Huh7 hepatocarcinoma cells stably expressing siRNA against control scrambled sequence (siNS) or TM4SF5 sequences (#7 and 8 sequences in Table 1, C-F) were processed to immunoblots and qRT-PCR (C), or seahorse analysis (D) for the ATP production rates or for ATP rate index (E to G, mitochondrial ATP production/glycolytic ATP production). Data shown represent three independent experiments. See also Figure 2.

Figure S3.


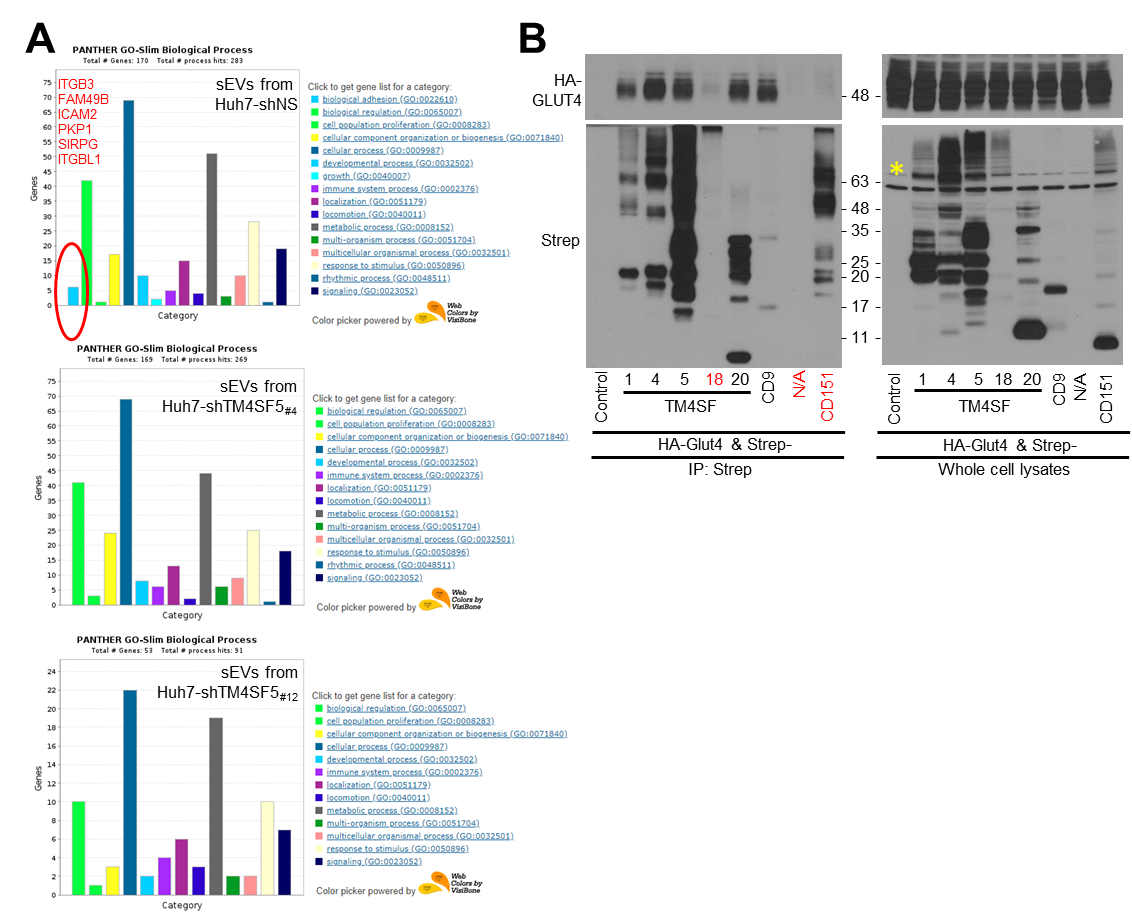


**Figure S3. MALDI-TOF analysis of sEVs from Huh7 cells without or with TM4SF5 suppression, and TM4SF5 binding to GLUT4. (A)** The sEVs were prepared from TM4SF5-positive Huh7 cells (transfected with shNS, Top in A) or TM4SF5-suppressed cells (with transfection of shTM4SF5 (against #4 or #12 sequences in Table 1: A, middle and bottom), before processing to MALDI-TOF proteomic analysis. Further, the proteins in the TM4SF5-positive or -negative sEVs were processed for PANDER (Protein Analysis Through Evolutionary Relationships, http://pantherdb.org) with Gene Ontology (GO) for biological process (PANDER-GO-slim). TM4SF5-posiotive sEVs showed additional proteins with red-highlighted names and circle (A, top). **(B)** HEK293FT cells were transfected with control or different plasmids for the indicated molecules for 48 h. N/A indicates untransfected cell lysates although transfection has been performed. Whole cell lysates were precipitated for Strep-tagged molecules prior to blotting for HA-GLUT4. See also Figure 3.

Figure S4.


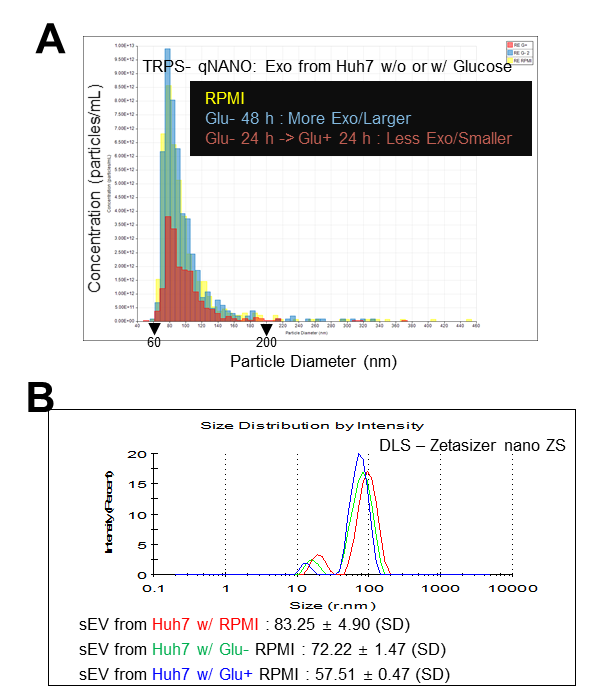


**Figure S4. Sizes in diameters of sEVs from Huh7 hepatocytes were increased by glucose-starvation, which were reduced by glucose repletion. (A)** sEVs or exosomes (Exo) from Huh7 hepatocytes with glucose depletion (w/o, for 48 h) or 24 h-repletion (following 24 h-depletion, w/) were analyzed for their size distribution in particle diameters (nm) by turnable resistive pulse sensing (TRPS). **(B)** Purified huh7 sEVs after size-exclusion chromatography in complete media (w/ RPMI), glucose-starved (w/ Glu-), or glucose-repleted (w/ Glu+) were analyzed by nanoparticle tracking analysis (NTA). Data shown represent three independent experiments. See also Figure 4.

Figure S5

**
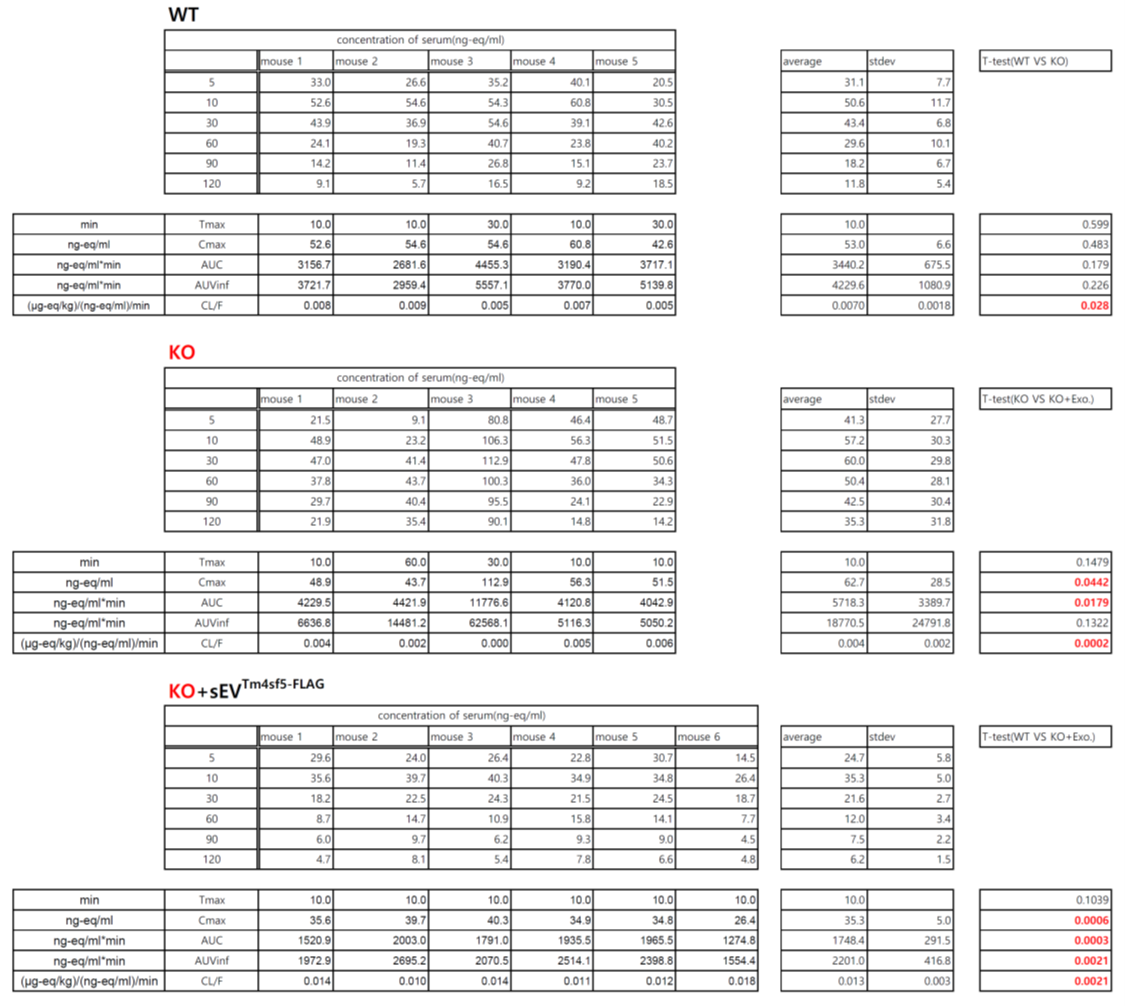
**

**Figure S5. Clearance of blood ^14^C-glucose intraperitoneally injected into the KO mice was improved by liv-sEV^Tm4sf5-FLAG^ injection.** Age-matched WT and KO male C57BL/6N mice (n=5) without any sEV pre-injection or KO mice (n=6) with a tail-vein-injection of liv-sEV^Tm4sf5-FLAG^ were intraperitoneally injected with ^14^C-glucose (40 μCi/kg). At several time points (5, 10, 30, 60, 90, and 120 min) after glucose injection, retro-orbital blood samples were processed for the LSC counting, to observe clearance of blood ^14^C-glucose depending on Tm4sf5 expression and liv-sEV^Tm4sf5-FLAG^ administration. See also Figure 6.

Figure S6.

**
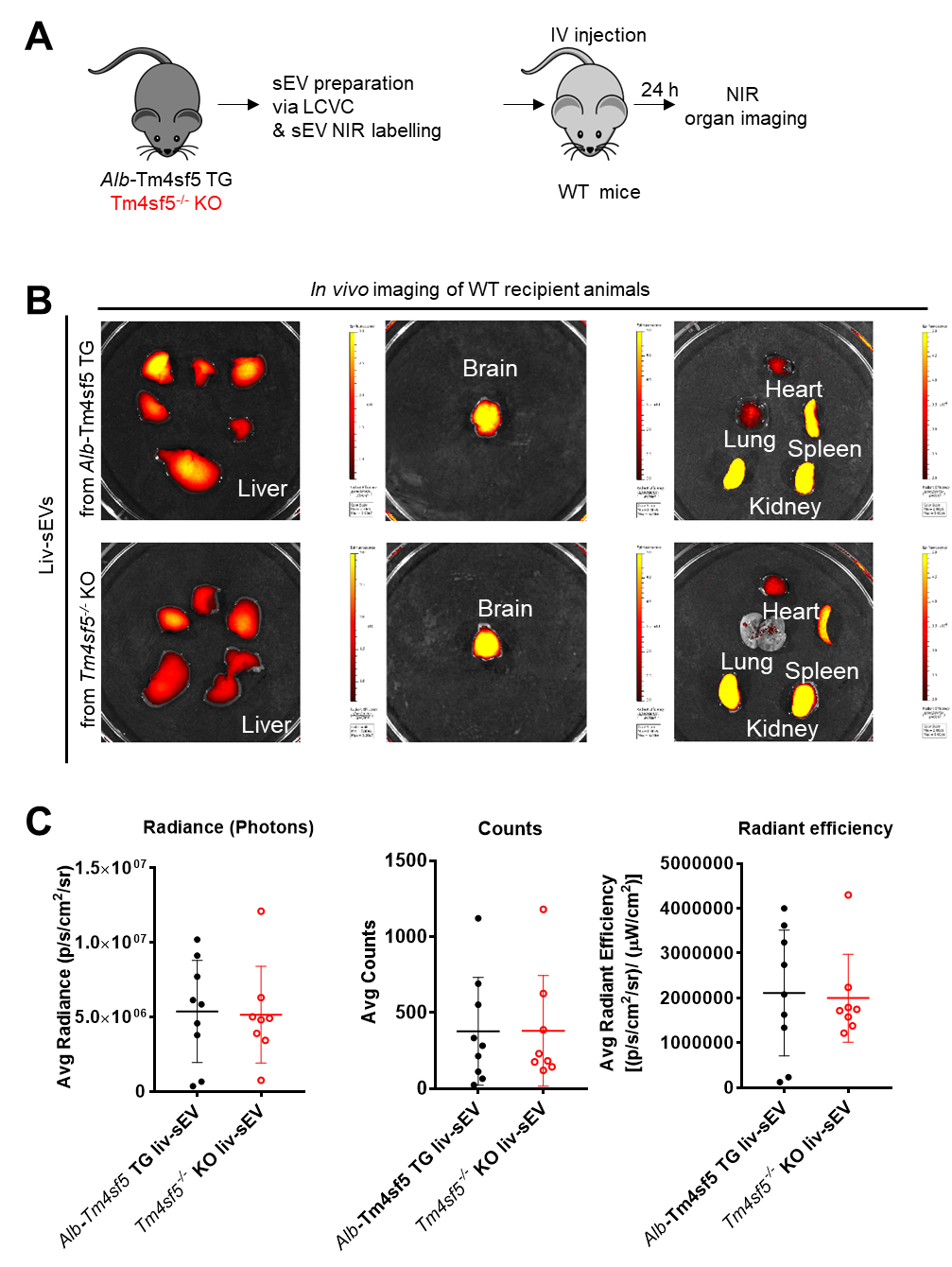
**

**Figure S6. *In vivo* analysis of WT mice intravenously injected with labeled-liv-sEVs from either *Tm4sf5* TG or KO mice. (A to C)** Near-infrared-dye-labeled liv-sEVs purified via the liver-closed vein circuit (LCVC) using *Tm4sf5^-/-^* KO or (*Alb*-Tm4sf5) TG mice were intravenously injected into the WT mice (n=10). One day later, the signals for sEVs in different organs were measured using intravital *in vivo* imaging system (A and B). The fluorescent signals for the sEVs incorporated into BAT were compared. See also Figure 6.

Figure S7

**Figure S7. Less supports for hepatocyte proliferation rate by sEVs-depleted or sEV/glucose-depleted FBS.** Huh7 or AML12 cells (5 x 10^4^ cells/well) were seeded in either normal 10% FBS-, UF-FBS, or d/UF-FBS and counted again after the indicated times. UF-FBS was prepared to deplete sEVs in the serum and d/UF-FBS was to remove the glucose in the UF-FBS, as explained in the Materials and Methods. The 24-hour proliferation rate was calculated and the rates of UF- and d/UF-treated cells were normalized to its control (i.e., cultured in normal sEVs-containing FBS). Statistics comparisons performed by two-way ANOVA. **, ***, or **** depict *P*<0.01, 0.001, or 0.0001 for statistical significance. See also Figure 6.
